# Neurons upregulate PD-L1 via IFN/STAT1/IRF1 to alleviate damage by CD8^+^ T cells in cerebral malaria

**DOI:** 10.1186/s12974-024-03114-7

**Published:** 2024-05-07

**Authors:** Yi Wang, Yan Shen, Jiao Liang, Shubiao Wang, Yuxiao Huang, Qinghao Zhu, Xizhi Zhang, Kangjie Yu, Guodong Tong, Chao Yang, Yinghui Li, Jun Wang, Ya Zhao

**Affiliations:** 1 Department of Medical Microbiology and Parasitology, Air Force Medical University, 169# Changle West Road, Xi’an, 710032 China; 2 Grade 2020 Clinical Medicine (Five-Year Program), Basic Medical College, Air Force Medical University, Xi’an, 710032 Shaanxi China; 3 Grade 2019 Clinical Medicine (Five-Year Program), Basic Medical College, Air Force Medical University, Xi’an, 710032 Shaanxi China; 4 Department of Pathology, Air Force Hospital of Eastern Theater, Nanjing, Jiangsu China; 5 College of Life Sciences, Northwest University, Xi’an, 710069 Shaanxi China

**Keywords:** Experimental cerebral malaria, Neuron, CD8^+^ T cell, PD-L1, Interferon

## Abstract

**Background:**

Cerebral malaria (CM) is the most lethal complication of malaria, and survivors usually endure neurological sequelae. Notably, the cytotoxic effect of infiltrating *Plasmodium*-activated CD8^+^ T cells on cerebral microvasculature endothelial cells is a prominent feature of the experimental CM (ECM) model with blood–brain barrier disruption. However, the damage effect of CD8^+^ T cells infiltrating the brain parenchyma on neurons remains unclear. Based on the immunosuppressive effect of the PD-1/PD-L1 pathway on T cells, our previous study demonstrated that the systemic upregulation of PD-L1 to inhibit CD8^+^ T cell function could effectively alleviate the symptoms of ECM mice. However, it has not been reported whether neurons can suppress the pathogenic effect of CD8^+^ T cells through the PD-1/PD-L1 negative immunomodulatory pathway. As the important inflammatory factor of CM, interferons can induce the expression of PD-L1 via different molecular mechanisms according to the neuro-immune microenvironment. Therefore, this study aimed to investigate the direct interaction between CD8^+^ T cells and neurons, as well as the mechanism of neurons to alleviate the pathogenic effect of CD8^+^ T cells through up-regulating PD-L1 induced by IFNs.

**Methods:**

Using the ECM model of C57BL/6J mice infected with *Plasmodium berghei* ANKA (PbA), morphological observations were conducted in vivo by electron microscope and IF staining. The interaction between the ECM CD8^+^ T cells (immune magnetic bead sorting from spleen of ECM mice) and primary cultured cortical neurons in vitro was observed by IF staining and time-lapse photography. RNA-seq was performed to analyze the signaling pathway of PD-L1 upregulation in neurons induced by IFNβ or IFNγ, and verified through q-PCR, WB, IF staining, and flow cytometry both in vitro and in vivo using IFNAR or IFNGR gene knockout mice. The protective effect of adenovirus-mediated PD-L1 IgGFc fusion protein expression was verified in ECM mice with brain stereotaxic injection in vivo and in primary cultured neurons via viral infection in vitro.

**Results:**

In vivo, ECM mice showed infiltration of activated CD8^+^ T cells and neuronal injury in the brain parenchyma. In vitro, ECM CD8^+^ T cells were in direct contact with neurons and induced axonal damage, as an active behavior. The PD-L1 protein level was elevated in neurons of ECM mice and in primary cultured neurons induced by IFNβ, IFNγ, or ECM CD8^+^ T cells in vitro. Furthermore, the IFNβ or IFNγ induced neuronal expression of PD-L1 was mediated by increasing STAT1/IRF1 pathway via IFN receptors. The increase of PD-L1 expression in neurons during PbA infection was weakened after deleting the IFNAR or IFNGR. Increased PD-L1 expression by adenovirus partially protected neurons from CD8^+^ T cell-mediated damage both in vitro and in vivo.

**Conclusion:**

Our study demonstrates that both type I and type II IFNs can induce neurons to upregulate PD-L1 via the STAT1/IRF1 pathway mediated by IFN receptors to protect against activated CD8^+^ T cell-mediated damage, providing a targeted pathway to alleviate neuroinflammation during ECM.

**Graphical Abstract:**

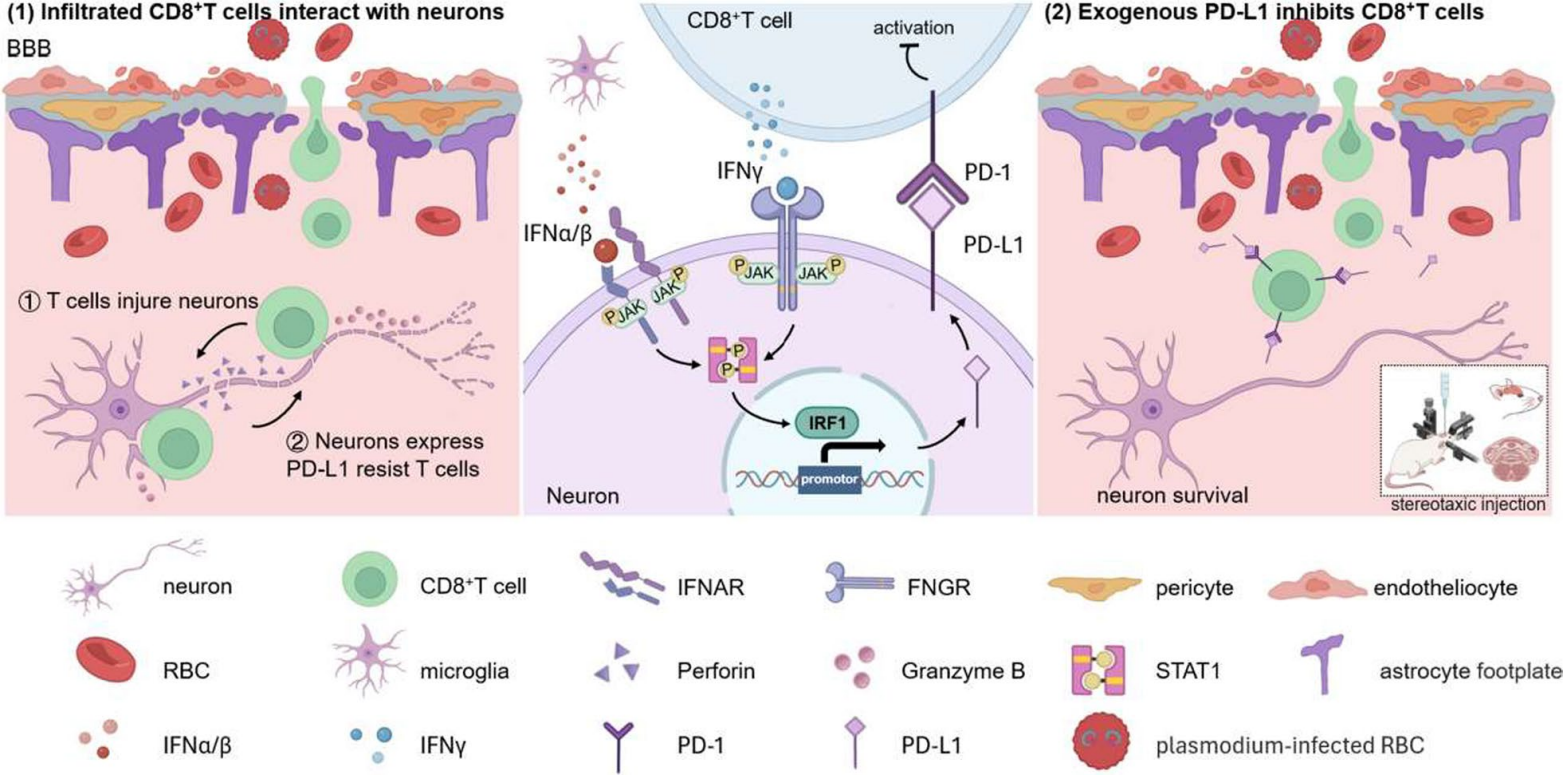

**Supplementary Information:**

The online version contains supplementary material available at 10.1186/s12974-024-03114-7.

## Background

Malaria is a severe infectious disease caused by the parasitic protozoa of the *Plasmodium* genus; it caused 249,000,000 cases and 608,000 deaths globally in 2022 [1–3]. Cerebral malaria (CM) is a lethal complication of *Plasmodium falciparum* infection, with high mortality (18–21%) and central nervous system (CNS) symptoms [4–8]. The intravascular sequestration of parasite-infected red blood cells (pRBCs) in the brain resulting in immunopathological damage is the primary cause of blood–brain barrier (BBB) disruption, and the most concerned is the cytotoxic effect of parasite-specific CD8^+^ T cells targeting brain microvascular endothelial cells [9–12]. Aging research suggested the clonal proliferation of T cells can be stimulated by antigens in the brain [13]. Our research has found that infiltrating CD8^+^ T cells showed proliferation in the brainstem of ECM mice [14], implying a potential direct interaction between CD8^+^ T cells and neurons in cerebral malaria.

The CD8^+^ T cells infiltrating the brain parenchyma of ECM mice expressed the immune checkpoint molecule programmed death-1 (PD-1), accompanied by injured neurons [15, 16]. The PD-1 and its ligand, programmed death-ligand 1 (PD-L1), can effectively inhibit the cytotoxic effect of CD8^+^ T cells, which is one of the most important pathways for enhancing anti-cancer effects [17] or immunotherapy effect on autoimmune diseases [18, 19]. Our group has confirmed that early intravenous injection of the PD-L1 fusion protein in mice inhibited CD8^+^ T cell function and enhanced the survival rate of ECM mice [20]. However, how the brain-infiltrating CD8^+^ T cells cause neuronal damage and whether neurons can increase PD-L1 expression to inhibit CD8^+^ T cell-mediated cytotoxicity have not been fully investigated.

Interferon γ (IFNγ) is the most important T cell-derived cytokine, which can mediate the adaptive expression of PD-L1, while the molecular mechanism depends on the immune microenvironment of different diseases. It was found that IFNγ-induced PD-L1 expression was mediated by IRF1 [21] in the human lung cancer cell line A549, NF-kB in melanoma [22], PI3K/mTOR pathway in glioma [23], STAT3 in T lymphoma [24], and JAK/STAT1 in hematopoietic tumor cells [25, 26], respectively. However, there are a few studies on the induction of PD-L1 expression by type I IFNs (IFNα/IFNβ), and the study on EAE has shown that IFNβ can induce neurons to activate FOXA1 through the PI3K/AKT pathway to promote the PD-L1 expression [27]. Consequently, this study aimed to elucidate the interaction between CD8^+^ T cells and neurons, as well as the mechanism of neurons to alleviate the pathogenic effects of CD8^+^ T cells through up-regulating PD-L1 induced by IFNs.

## Methods

### Ethics statement

The Institutional Review Board of the Air Force Medical University (No. IACUC-20200407) approved animal experiments. All procedures were performed to minimize injury to the experimental mice.

### Mice and infection

C57BL/6J mice were procured from the Experimental Animal Center of the Air Force Medical University. *Ifnar1*^−/−^ C57BL/6N and *Ifngr1*^−/−^ C57BL/6J mice were separately obtained from Cyagen Bioscience (China) and Shanghai Model Organisms Center (China). The mice feeding system was of a specific pathogen-free grade, comprising adequate water and food. The *Plasmodium berghei* ANKA (PbA) strain was preserved in liquid nitrogen, maintained regularly, and used as previously reported [16]. Male mice aged 5–6 weeks (18–20 g in weight) were injected intraperitoneally (i.p.) with 5 × 10^6^ pRBCs to induce ECM. The control group received no treatment, and all groups were fed normally. On day 7 after infection, the mice were sacrificed, and their whole brains were collected after heart perfusion with normal saline for subsequent experimental processing.

### Antibodies and reagents

The primary antibodies against mouse CD8 (GB13429) and Ki67 (GB111141) were purchased from ServiceBio (China). The antibodies against mouse PD-L1/CD274 (66248-1-Ig), NeuN (66836-1-Ig), MAP2 (17490-1-AP), CD18 (10554-1-AP), and APP (25524-1-AP) were purchased from Proteintech (China). Antibodies against p-STAT1 (ET1611-20), LC3 (ER1802-2), and HLA Class 1 ABC (EM1801-10) were purchased from HUABIO (China), and IRF1 (CY5507) from Abways (China), respectively. Antibodies against IFNβ (AB218229) and IFNγ (A12450) were purchased from Abcam (UK) and ABclonal (China), respectively. Antibody against β-actin (CW0096) and horseradish peroxidase-conjugated secondary antibodies were purchased from CWBio (China), including goat anti-rabbit IgG (CW0103S) and goat anti-mouse IgG (CW0102S). Fluorescence-labeled secondary antibodies were purchased from ServiceBio (China) for immunofluorescence staining, including FITC-conjugated goat anti-mouse IgG (GB22301), FITC-conjugated goat anti-rabbit IgG (GB22303), Cy3-conjugated goat anti-mouse IgG (GB21301), and Cy3-conjugated goat anti-rabbit IgG (GB21303). PE-labeled antibody against mouse CD274 (B7 homolog 1, PD-L1) (124307) and FITC-labeled H-2 Kb/H-2Db (114605) for flow cytometry were purchased from BioLegend (USA). IFNγ (50709-MNAH) was purchased from SinoBiological (China) and IFNβ (I9032-1VL) was from Sigma-Aldrich (USA). Fludarabine (HY-B0069) was purchased from MedChemExpress (USA).

### Transmission electron microscopy

Mice with neurological symptoms were anesthetized and sacrificed on day 7 after PbA infection, and their brainstems were isolated immediately. The brainstems were fixed with 2.5% glutaraldehyde (P1126, Solarbio, China) at 4 °C for 2 h and then washed several times with phosphate-buffered saline (PBS) after fixation. The tissues were progressively dehydrated with 50%, 75%, 95%, and 100% ethanol, embedded in epoxy resin, and cut into 0.5 μm sections with an ultra-microtome for electron microscopy (JEM-1400PLUS, JEOL, Japan).

### Histology

Mice were perfused with normal saline through the heart, and their whole brains were fixed in 4% paraformaldehyde (PFA) overnight. The brain tissues were cut into 5 μm sections after dehydration, transparency, and paraffin embedding, and then collected on slides. After deparaffinization and rehydration, these sections were used for hematoxylin and eosin (H&E), Nissl, immunohistochemistry, and immunofluorescent staining.

### Immunohistochemistry staining

Paraffin-embedded tissue sections were prepared in advance, and antigen retrieval was completed with citrate antigen retrieval solution (E673001, BBI, China). The sections were sequentially incubated with 3% hydrogen peroxide (H_2_O_2_) for 10 min, blocking buffer for 30 min (with 2% bovine serum, 3% bovine serum albumin, and 0.2% Triton X-100), and primary antibodies overnight at 4 °C. The sections were subsequently incubated with secondary antibodies for 1 h at room temperature after rinsing in PBS with 0.2% Triton X-100 (PBST). The sections were rapidly incubated for staining with 3,3-diaminobenzidine (DAB) (A690009-0025, BBI, China). The hematoxylin (E607317-0100, BBI) was used for counterstaining. The Pannoramic DESK (P-MIDI, P25, Japan) and CaseViewer 2.4 (3DHISTECH, Hungary) were used for digital slide scanning and image acquisition, respectively. The images were analyzed using ImageJ 1.53c.

### Immunofluorescent staining

Paraffin-embedded tissue sections of mouse brains were prepared as described above. Neurons were seeded and cultured on coverslips in 24-well plates coated with poly l-lysine (25988-63-0, Sigma, USA). After various cell treatments, the coverslips were fixed in 4% PFA for 20 min. Samples on coverslips and sections on slides were sequentially incubated in a blocking buffer for 30 min, primary antibodies overnight, and secondary antibodies for 2 h. Notably, all antibodies were diluted in a blocking buffer, as mentioned above. The coverslips and sections were mounted in a mounting medium with DAPI (ab104139, Abcam, UK). Image scanning and analysis were performed as previously described.

### Primary murine cortical neuron culture

The brains of mice were aseptically collected within 24 h after birth and placed in cold D-Hank’s balanced salt solution. The cerebral cortices were isolated under a microscope, and the meninges and blood vessels were stripped as much as possible. The cortices were cut and digested at 37 °C for 10 min with 0.25% trypsin (SH30042.01, Hyclone, USA). The digestion was terminated by adding Dulbecco’s modified eagle medium/Ham’s F-12 (DMEM-F12) (31330-038, Gibco, USA) supplemented with 10% fetal bovine serum (FBS) (16140063, Gibco, USA). Single cells were mechanically separated by the repeated blowing and sucking of the tissue block using a Pasteur pipette, centrifuged, and resuspended in Neurobasal A medium with 2% B-27 supplement (17504-044, Gibco, USA), 2 mM Glutamine (25030-164, Gibco, USA), and 0.5% penicillin and streptomycin (14140-148, Gibco, USA). The cells were seeded on poly l-lysine coated plates and cultured in a 5% carbon dioxide incubator at 37 °C. Neurons were mature for subsequent experiments approximately after 2 weeks, with 50% of the medium renewed every 3 days.

### Purification of spleen CD8^+^ T cells

Mice were anesthetized and sacrificed to isolate intact spleens. The spleens were ground in cold PBS and passed through a 200-mesh metal sieve to prepare a tissue suspension. The suspension was then centrifuged, and the precipitate was resuspended with erythrocyte lysate (AR1118, Bosterbio, China). The magnetic bead sorting kit (558471, BD, USA) (negative selection) was used to isolate CD8^+^ T cells from the suspension. Naïve and ECM CD8^+^ T cells were obtained from control mice and mice infected with PbA for 5–8 days, respectively.

### Cells co-culture and live-cell time-lapse photography

ECM-activated CD8^+^ T cells and mature neurons were co-cultured at an effector-to-target cell ratio of 2:1 to 5:1 for 24 h. Then, total RNA, proteins, and supernatants were separated and collected for subsequent tests. CD8^+^ T cells used for cell adhesion experiments were isolated from wild-type or GFP transgenic mice. The EVOS M7000 imaging system (Thermo Fisher, USA) was used for real-time live cell imaging, automatically taking pictures every 30 s for 4 h. Notably, all pictures were combined into a short video using Microsoft Clipchamp.

### Western blot analysis

The olfactory bulb, cerebrum, cerebellum, and brainstem were isolated from the mice separately. Proteins were extracted using RIPA assay lysis buffer (AR0102, Bosterbio, China) with 1% protease inhibitor cocktail (4693116001, Roche, Switzerland). A protein concentration assay kit (AR0146, Bosterbio, China) was used to determine the protein concentration. Proteins were separated on 4–20% BeyoGel™ Plus precast PAGE gels (P0523M, Beyotime, China) and then transferred to polyvinylidene fluoride membranes (ISEQ00010, Millipore, USA). The membranes were incubated in 5% skim milk for 1 h, with primary antibodies (diluted according to the instructions) and secondary antibodies. The membranes were washed thrice with PBST after incubation with antibodies. A high-sensitivity enhanced chemiluminescence ready-to-use substrate kit (AR1197, Bosterbio, China) was used to detect protein bands. The gray values of bands were measured using ImageJ 1.53c to analyze the relative expression of the target protein, using β-actin as an endogenous reference.

### Quantitative real-time PCR

Total RNA was extracted using the TRIZOL reagent method and then reverse transcribed into cDNA using reverse transcription supermix (R222-01, Vazyme, China). Quantitative real-time PCR (q-PCR) was conducted using 2× SYBR qPCR master mix (Q311-02, Vazyme, China). The primers (Table S1) were synthesized by Sangon Biotechnology Co. (China). Relative gene expression was calculated using the 2^−ΔΔCT^ method with β-actin as an endogenous reference.

### Flow cytometry

For cell damage detection, neurons were stained for 20 min with 1 µM SYTOX™ green (S7020, Invitrogen, USA), 100 µg/mL propidium iodide (PI) nucleic acid stain (ST511, Beyotime, China), or 2 μM JC-1 (HY-K0601, MCE, China). After washing with PBS, the neurons were digested with 0.25% trypsin, centrifuged, and resuspended in D-Hank’s solution with 0.5% FBS. To detect PD-L1^+^ cells, neurons were incubated with the above PE-labeled anti-PD-L1 antibody for 30 min and then resuspended in D-Hank’s solution. The cells were passed through a 70 μm strainer to avoid cell clumps before detection using a flow cytometer (FACSCanto, BD, USA). Each sample collected contained 2 × 10^4^–20 × 10^4^ cells. Data were analyzed using FlowJo 7.6.1.

### LDH release test

Spleen CD8^+^ T cells or T cell culture supernatants were separately added to the neuronal culture system, and the supernatants were collected after co-culture for 24 h. Neuronal damage was detected using an LDH cytotoxicity assay kit (C0016, Beyotime, China). Absorbance was measured at 490 nm with a microplate reader (Model 680, Bio-Rad, USA) and was positively correlated with LDH activity.

### Single-cell RNA-sequencing analysis

The brainstems of mice were collected and dissociated into single cells after heart perfusion with normal saline. Single-cell suspensions were loaded onto a 10X Genomics Chromium instrument following the manufacturer’s instructions for the 10X Genomics Chromium Single-Cell 3′ kit (V3). The following cDNA amplification and library construction steps were performed according to the standard protocol. Libraries were sequenced on an Illumina NovaSeq 6000 sequencing system by LC-Bio Technology Co. Ltd. (China) at a minimum depth of 20,000 reads per cell. A total of 18,647 transcriptomes from single cells in the brainstem of 3 ECM mice and 2 healthy mice passed the quality control threshold. Cell types were inferred using marker genes identified from literatures and the web-based tool (http://xteam.xbio.top/CellMarker/). Data analysis and graphics generation were performed using the OmicStudio tools at https://www.omicstudio.cn/cell.

### RNA-sequencing analysis

Total RNA was extracted from neurons stimulated with IFNβ (100 U/mL), IFNγ (20 ng/mL), or ECM CD8^+^ T cells for 24 h and sent to LC-BIO Technology Co. Ltd. (China) for RNA-sequencing (RNA-seq). Each group comprised three samples under the same treatment conditions. The aforementioned OmicStudio tools were used for data analysis and plotting, including differential gene screening and Gene Ontology enrichment analysis.

### Stereo-localization and microinjection of brain

Mice were anesthetized, head-fixed, shaved, and disinfected for subsequent craniotomy and suture. The stereoscopic location of the injection site was determined using a brain atlas. Holes were drilled into the skull marks, microneedles were inserted according to the coordinates, and the virus was injected. The scalp was sutured, and the mice were put back in the cage. The mice were infected with PbA next day after microinjection, and were sacrificed on day 7 after infection. Brain tissue was collected for histological examination.

### Statistical analysis

Data were processed and analyzed with GraphPad Prism software version 9.0. Quantitative data were expressed as mean ± standard deviation. Two-group comparisons were performed using Student’s t-tests. Normal tests were performed before t-tests, and the sample data followed normal distribution. Statistical significance was set at *P* < 0.05.

## Results

### The damage of neuron is accompanied with activation of infiltrating CD8^+^ T cells in ECM brain

**Fig. 1:**
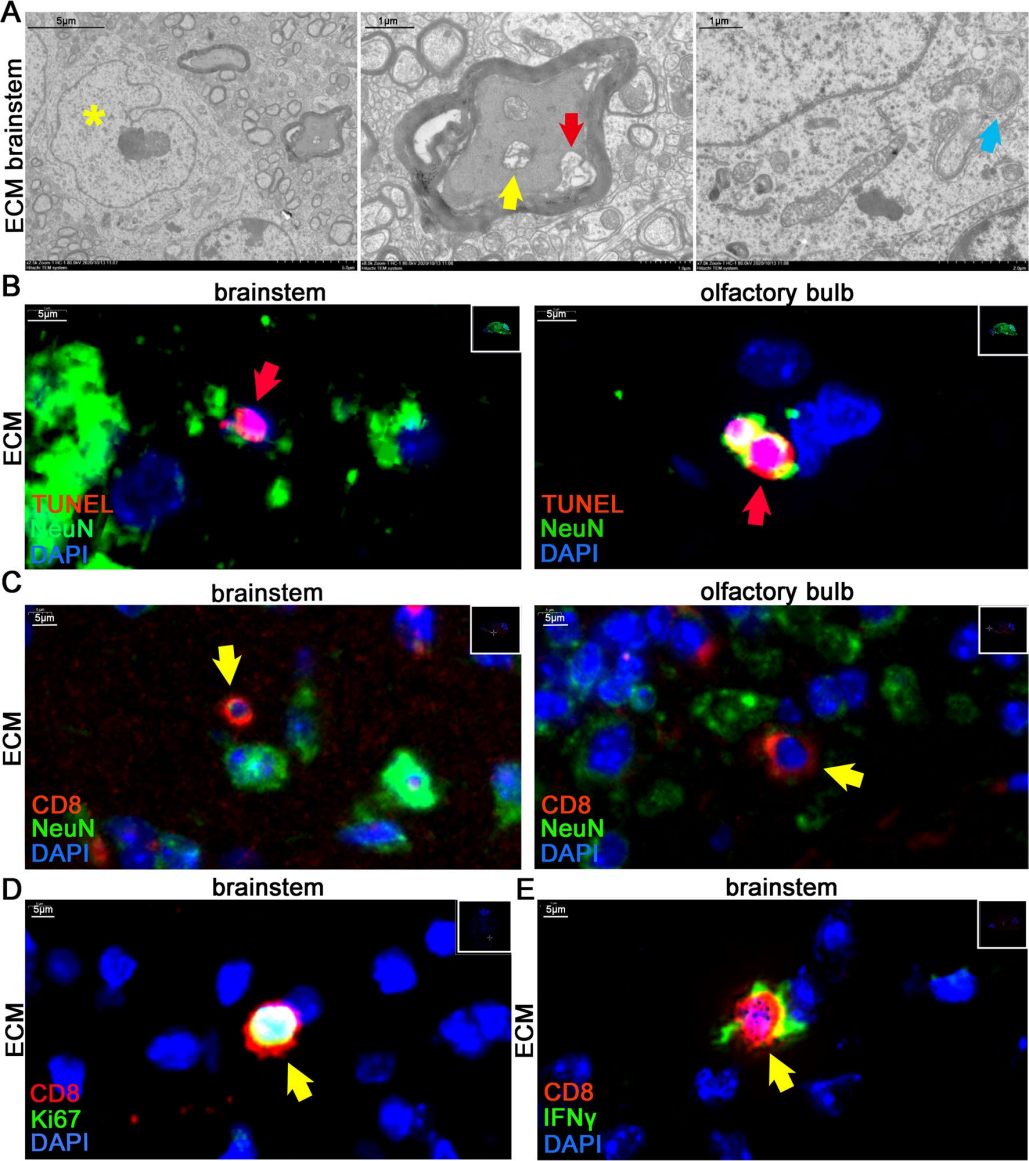
Neurological damage and activated CD8^+^ T cell infiltration in the brains of ECM mice. **A** Electron microscope observation of neuron in the brainstem of ECM mice (yellow asterisk: change in nucleus shape; red arrow: demyelination; yellow arrow: mitochondria damage; blue arrow: autophagosome). **B** IF staining of TUNEL^+^ neurons (red arrow) in the brainstem and olfactory bulb of ECM mice. **C** IF staining of infiltrating CD8^+^ T cells (yellow arrow) which are close to neurons (NeuN^+^) in the brainstem and olfactory bulb of ECM mice. **D** IF staining of Ki67^+^CD8^+^ T cells (yellow arrow) in the brainstem of the ECM mice. **E** IF staining of IFNγ^+^CD8^+^ T cells (yellow arrow) in the brainstem of ECM mice

Notably, some C57BL/6J mice showed neurological symptoms, such as torpor, lethargy, convulsions, and mania, on days 7–9 after PbA infection, indicating the occurrence of ECM. H&E staining revealed hemorrhagic spots in the olfactory bulb, cerebrum, cerebellum, and brainstem of ECM mice, with the brainstem exhibiting more severe hemorrhaging (Figure S1A). As the respiratory center, the injury of brainstem may be the direct cause of death in ECM mice, so follow-up studies mainly focus on the brainstem. Electron microscopy of the brainstem of ECM mice showed that the shape of the nucleus in neurons changed from spherical to irregular, with demyelination, autophagosome formation, and mitochondrial damage (Fig. 1A). Compared to control mice, ECM mice showed decreased levels of synaptophysin in the cerebrum (Figure S1B) and reduced Nissl bodies within the neurons in the brainstem (Figure S1C), indicating impaired neuronal function. Furthermore, there were few apoptotic cells (TUNEL^+^) in the brain of control mice, while in ECM mice, TUNEL^+^ cells were observed in entire brain parenchyma (Figure S1D), and the brainstem and olfactory bulb contained TUNEL^+^ neurons (Fig. 1B). Similarly, IF staining showed that the LC3 levels, an autophagy marker [28], were increased in neurons in the cerebrum of ECM mice (Figure S1E). These results indicated that nerve cells of ECM mice, especially neurons, could undergo different forms of cell damage and death, such as apoptosis, autophagy, and ferroptosis [29], which were closely associated with developing neurological symptoms during ECM.

In addition to nerve damage, we found infiltrating CD8^+^ T cells in the brainstem and olfactory bulb of ECM mice, which were spatially close to or directly in contact with neurons (Fig. 1C). Notably, most of these infiltrating CD8^+^ T cells showed proliferative activity (Ki67^+^) (Fig. 1D and S1F) and secreted IFNγ (Fig. 1E). This finding suggested that the direct contact and killing effects of activated CD8^+^ T cells on neurons may directly influence the extent of nerve injury.

### The ECM-activated CD8^+^ T cells exert cytotoxic effects on primary neurons through direct contact

**Fig. 2:**
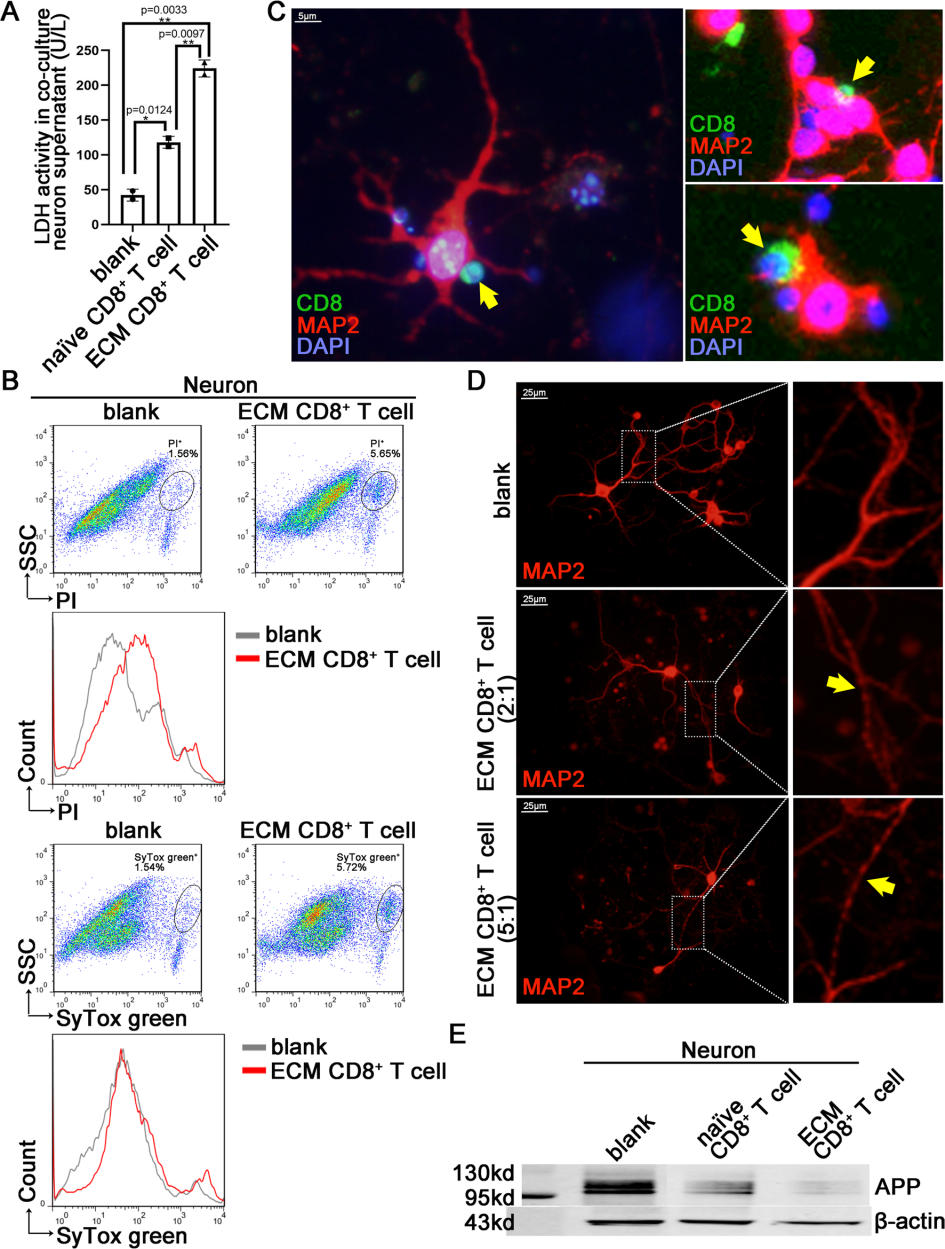
Direct interaction between neurons and ECM CD8^+^ T cells in the co-culture system. Primary cortical neurons were co-cultured with CD8^+^ T cells isolated from the spleen of ECM mice for 24 h. **A** LDH activity in supernatants of neurons co-cultured with ECM CD8^+^ T cells or naïve CD8^+^ T cells. Data are expressed as mean ± SD; unpaired t-test, n = 2 per group. **B** Flow cytometry of PI and SyTox green in neurons co-cultured with ECM CD8^+^ T cells. **C** IF staining of ECM CD8^+^ T cells (yellow arrow) adhesion to neurons (MAP2^+^). **D** IF staining of MAP2 in neurons co-cultured with different effect-target ratios of ECM CD8^+^ T cells (yellow arrow: axon damage). **E** WB analysis of APP levels in neurons co-cultured with ECM CD8^+^ T cells or naïve CD8^+^ T cells

During the co-culture of primary cultured mature mouse cortical neurons and CD8^+^ T cells, we observed that ECM CD8^+^ T cells could directly adhere to neurons and that the number and ratio of adhering CD8^+^ T cells were significantly higher than those of naïve CD8^+^ T cells (Figure S2A). Correspondingly, the LDH assay indicated more severe damage to neurons co-cultured with ECM CD8^+^ T cells (Fig. 2A). ECM CD8^+^ T cell culture supernatants were added to the neuron culture system, and the neuronal damage worsened as the proportion of supernatant increased by the cell counting kit-8 (CCK-8) assay (Figure S2B). Flow cytometry showed that the PI^+^ (cell death marker) neurons and SyTox green^+^ (cell membrane damage marker) neurons increased after ECM CD8^+^ T cell stimulation (Fig. 2B). Living cell JC-1 staining [30] revealed that ECM CD8^+^ T cell stimulation increased mitochondrial damage in neurons (Figure S2C). IF staining showed that CD8^+^ T cells were in close contact with neurons (Fig. 2C), and it further corroborated that the higher the CD8^+^ T cell-to-neuron effector-target ratio, the more serious the damage to axons according to neuron marker microtubule associated protein 2 (MAP2) (Fig. 2D and S2D). WB analysis revealed that compared with naïve CD8^+^ T cells, ECM CD8^+^ T cells significantly reduced the neuronal levels of amyloid-β precursor protein (APP) (Fig. 2E), which plays a physiological role in modulating synaptic transmission and plasticity [31]. These findings showed that the ECM CD8^+^ T cells actively adhered to the neurons and exerted neurotoxic effects.

Using time-lapse photography, it was directly observed that ECM CD8^+^ T cells adhered to the bodies and subsequently caused the death of neurons (Video 1), and they also actively adhered to and damaged axons (Video 2). Notably, we observed that ECM CD8^+^ T cells proliferate after interacting with neurons within a few hours (Video 3), and this implies that ECM CD8^+^ T cell proliferation is reactivated by interactions with neurons, such as antigen presentation. In addition, after 24 h of co-culture with ECM CD8^+^ T cells, the expression of the major histocompatibility complex class I (MHC-I) molecule *H2-D1* in neurons increased according to q-PCR assay (Figure S2E) and FC analysis of neurons also showed consistent results (Figure S2F). Using IF assay, we observed the polarization expression of the co-stimulatory cell-surface molecule lymphocyte function-associated antigen-1 (LFA-1, CD18) in ECM CD8^+^ T cells, which is involved in immune synapse formation to activate specific CD8^+^ T cell toxicity [32], and the MHC-I molecules H2-D/K were widely but weakly expressed in neurons relative to those in T cells (Figure S2G).

### Type I and type II IFNs induce neurons to up-regulate PD-L1 expression

**Fig. 3:**
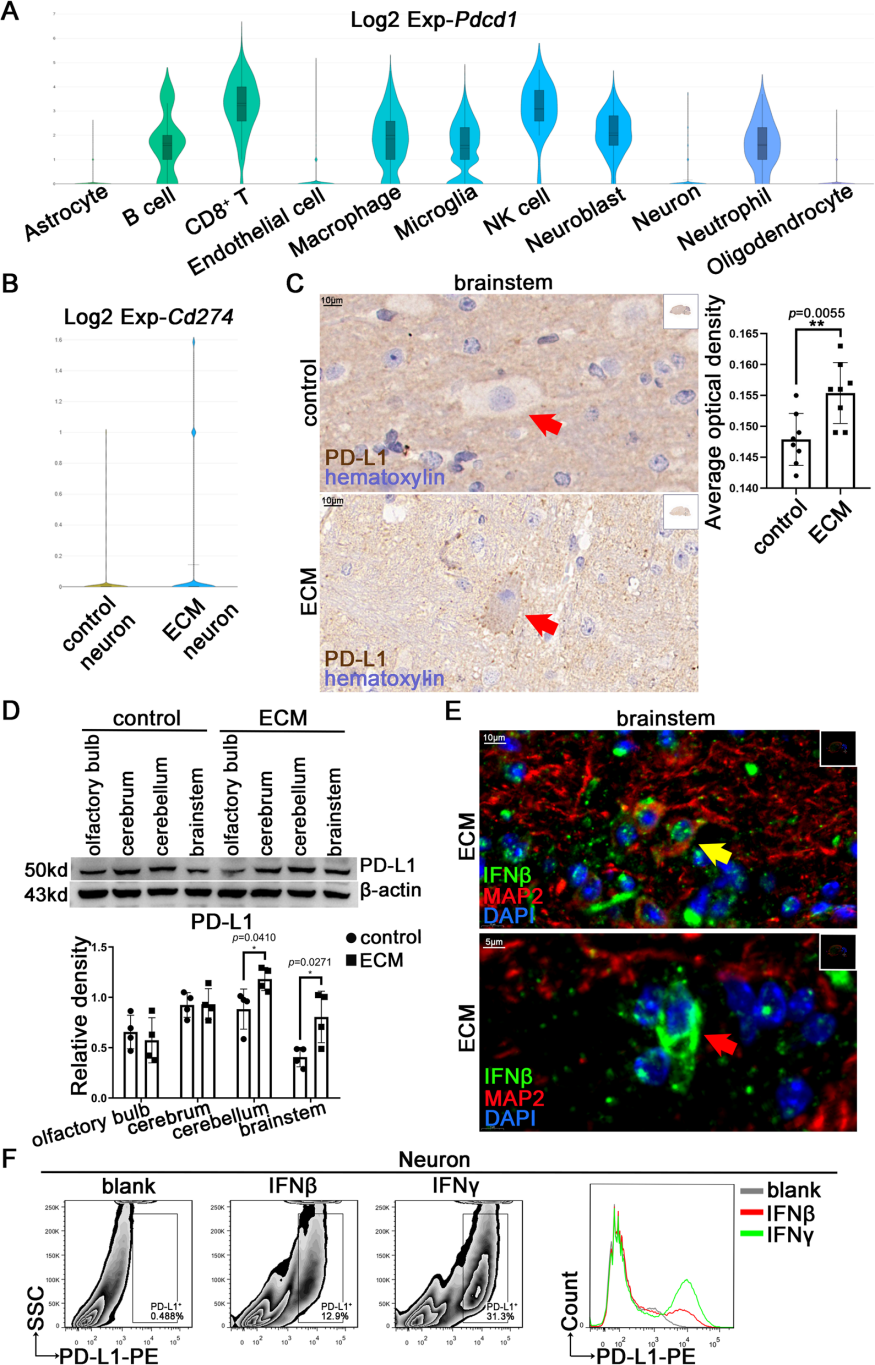
IFNβ or IFNγ induces nerve cells to upregulate PD-L1 during ECM. **A** The violin plot of *Pdcd1* expression in different types of cells from the brainstem of ECM mice (n = 3) through scRNA-seq analysis (*P*_adj_ < 0.05). **B** The violin plot of *Cd274* expression in neurons from the brainstem of control mice (n = 2) and ECM mice (n = 3) through scRNA-seq analysis (*P*_adj_ < 0.05). **C** IHC staining of PD-L1 levels in the brainstem of control and ECM mice (red arrow: nerve cell). Data are expressed as mean ± SD; unpaired t-test; n = 8 fields per group. **D** WB analysis of PD-L1 levels in the olfactory bulb, cerebrum, cerebellum, and brainstem of control and ECM mice. Data are expressed as mean ± SD; unpaired t-test; n = 4. **E** IF staining of the IFNβ secretion in neurons (yellow arrow) and MAP2^−^ cells (red arrow) in the brainstem of ECM mice. F) Flow cytometry of PD-L1 levels on neurons stimulated with IFNβ (100 U/mL) or IFNγ (20 ng/mL)

scRNA-seq analysis of the brainstem of ECM mice showed that the infiltrating CD8^+^ T cells highly expressed PD-1 coding gene *Pdcd1* (Fig. 3A), which had an immunosuppressive effect on the T cell activity mediated by PD-L1. Notably, scRNA-seq analysis also suggested that neurons can potentially express the PD-L1 gene, *Cd274* (Fig. 3B). Meanwhile, IHC staining showed that PD-L1 levels were extensively increased in the brain of ECM mice compared with those in control mice (Fig. 3C and S3A). Similarly, PD-L1 levels in the cerebellum and brainstem were substantially upregulated, as determined using WB (Fig. 3D), which could be induced by various inflammatory cytokines, including IFNβ and IFNγ [33, 34]. CD8^+^ T cells in the brains of ECM mice secreted IFNγ (Fig. 1E), and IF analysis revealed the expression of IFNβ in the brain, including various nerve cells (Fig. 3E), indicating that IFN signaling participated in the inflammatory microenvironment of the ECM brain.

Primary cortical neurons were individually stimulated with IFNβ or IFNγ to mimic the immune microenvironment of brain during ECM. q-PCR assay revealed that neurons upregulated the expression of *Cd274* upon IFNβ or IFNγ stimulation, and the *Cd274* expression varied over time, peaking at 5 h after IFNβ or IFNγ stimulation followed by a gradual decline (Figure S3B). WB analysis showed that the PD-L1 levels in neurons increased continuously with IFNβ stimulation (Figure S3C). IF staining revealed elevated PD-L1 levels in neurons with IFNβ or IFNγ stimulation for 24 h (Figure S3D), and the FC detection confirmed the same changes (Fig. 3F). These results suggested that IFNs could induce neurons to upregulate PD-L1 to inhibit T cell activity. However, the underlying molecular mechanism still needs to be elucidated.

### IFNs induce expression of PD-L1 in neurons through the STAT1/IRF1 pathway

**Fig. 4:**
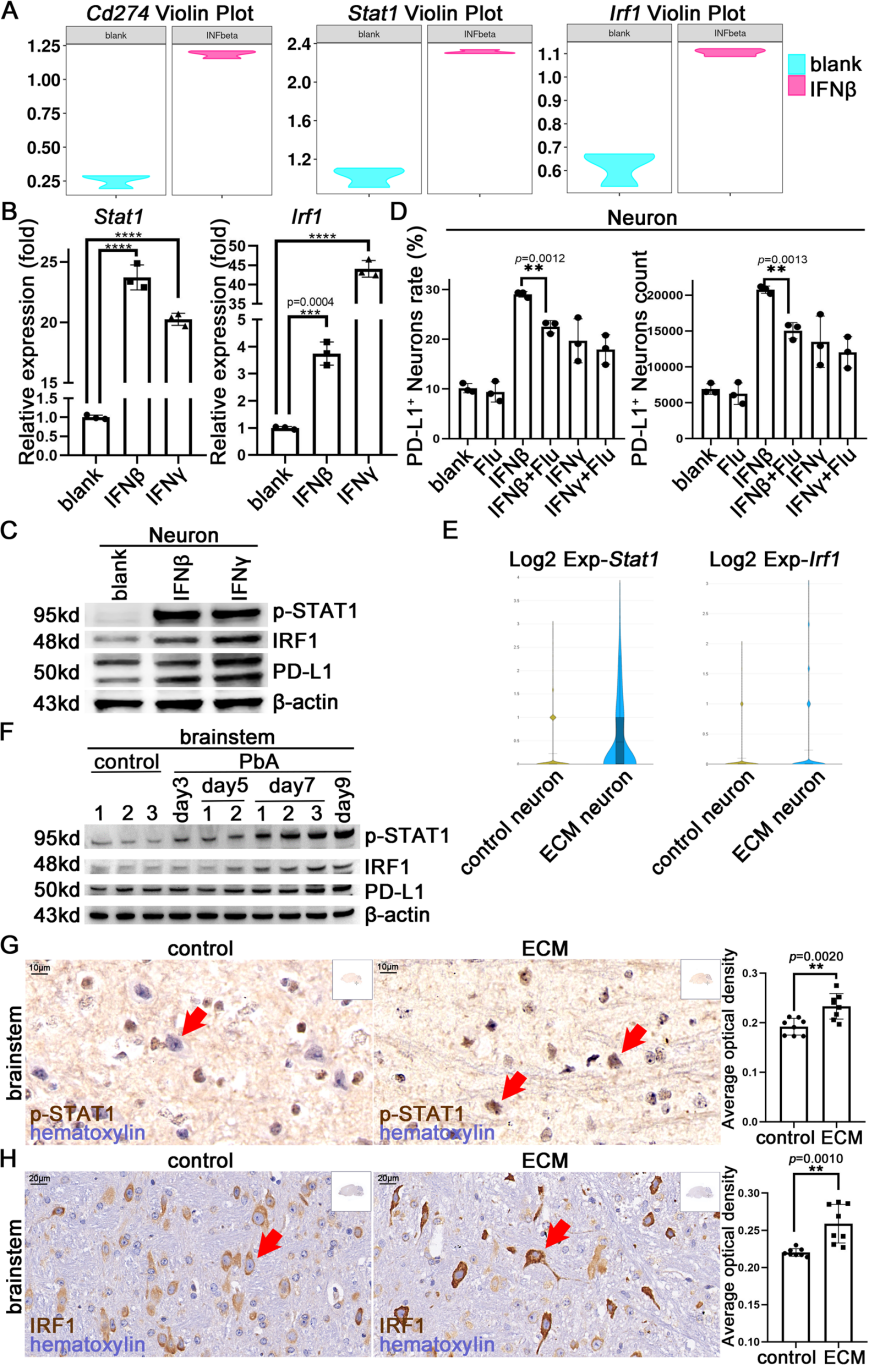
IFNβ or IFNγ induces PD-L1 expression in neurons through the STAT1/IRF1 pathway. **A** Violin plots of *Cd274*, *Stat1*, and *Irf1* expression in neurons stimulated with IFNβ through RNA-seq analysis (n = 3 per group) (*P*_adj_ < 0.05). **B** q-PCR detection of *Stat1* and *Irf1* expression in neurons with IFNβ (100 U/mL, the same below) or IFNγ (20 ng/mL, the same below) stimulation. Data are expressed as mean ± SD; unpaired t-test, n = 3 per group, ****P < 0.0001. **C** WB analysis of p-STAT1, IRF1, and PD-L1 levels in neurons with IFNβ or IFNγ stimulation. **D** Flow cytometry of PD-L1 levels on neurons treated with fludarabine (20 µM), IFNβ, or IFNγ. **E** Violin plots of *Stat1* and *Irf1* expression in neurons in the brainstem of control mice (n = 2) and ECM mice (n = 3) through scRNA-seq analysis (*P*_adj_ < 0.05). **F** WB analysis of p-STAT1, IRF1, and PD-L1 levels in the brainstem of mice with PbA infection on days 3, 5, 7, and 9. **G** IHC staining of p-STAT1 in the brainstem of control and ECM mice (red arrow: neuron). **H** IHC staining of IRF1 in the brainstem of control and ECM mice (red arrow: neuron). **G**, **H** Data are expressed as mean ± SD; unpaired t-test; n = 8 fields per group

RNA-seq was performed on neurons stimulated with IFNβ, IFNγ, or ECM CD8^+^ T cells for 24 h, with untreated neurons as control, to accurately identify the key signaling pathways and molecules involved in regulating PD-L1 expression. Gene Ontology enrichment analysis revealed that multiple IFN-related pathways were significantly activated in neurons stimulated by ECM CD8^+^ T cells (Figure S4A). Violin plots showed that *Cd274*, *Stat1*, and *Irf1* were upregulated in neurons with IFNβ (Fig. 4A), IFNγ, or CD8^+^ T cells stimulation (Figure S4B). Similarly, q-PCR analysis revealed that neurons upregulated *Stat1* and *Irf1* expression with IFNβ or IFNγ stimulation (Fig. 4B). The *Stat1* expression increased continuously in response to IFNβ stimulation, while it peaked at 10 h under IFNγ stimulation (Figure S4C). The *Irf1* expression peaked at 2 h under IFNβ or IFNγ stimulation and gradually decreased (Figure S4C). WB results confirmed that neurons stimulated with IFNβ or IFNγ showed increased levels of p-STAT1, IRF1, and PD-L1 (Fig. 4C), and the p-STAT1 increased over time (Figure S4D). Moreover, IF staining of p-STAT1 in neurons confirmed similar changes (Figure S4E).

In addition, the STAT1 phosphorylation inhibitor, fludarabine [35], could partially inhibit the upregulation of the *Stat1* in neurons individually stimulated by IFNβ or IFNγ, and could partially inhibit the upregulation of *Cd274* stimulated by IFNγ and *Irf1* by IFNβ (Figure S4F). FC detection showed that fludarabine reduced the upregulation of the proportion and number of PD-L1^+^ neurons in response to IFNβ stimulation but did not significantly affect IFNγ stimulation (Fig. 4D and S4G), which differs from the q-PCR result, suggesting an inconsistency between the protein and transcription levels. Fludarabine, at concentrations starting from 5 µmol/L, inhibited the upregulation of IRF1 and PD-L1 levels in neurons stimulated with IFNγ, as measured using WB (Figure S4H). However, the stimulation effect of IFNγ may be enhanced by the solvent dimethyl sulfoxide (DMSO) in the low-dose group (Figure S4H).

scRNA-seq analysis suggested that neurons highly expressed *Stat1* and *Irf1* in the brainstem of ECM mice (Fig. 4E). WB analysis of mouse brains revealed higher levels of p-STAT1, IRF1, and PD-L1 in the brainstem of ECM mice, which increased with infection time (Fig. 4F). IHC staining showed that the neurons in the brainstem of ECM mice highly expressed p-STAT1 compared with that in control mice (Fig. 4G). IRF1 levels of neurons in the brainstem remarkably increased based on IHC staining, whereas other brain regions showed no significant changes (Fig. 4H and S4I).

### IFNAR or IFNGR deletion prevents the upregulation of PD-L1 in the brain of mice infected with PbA

**Fig. 5:**
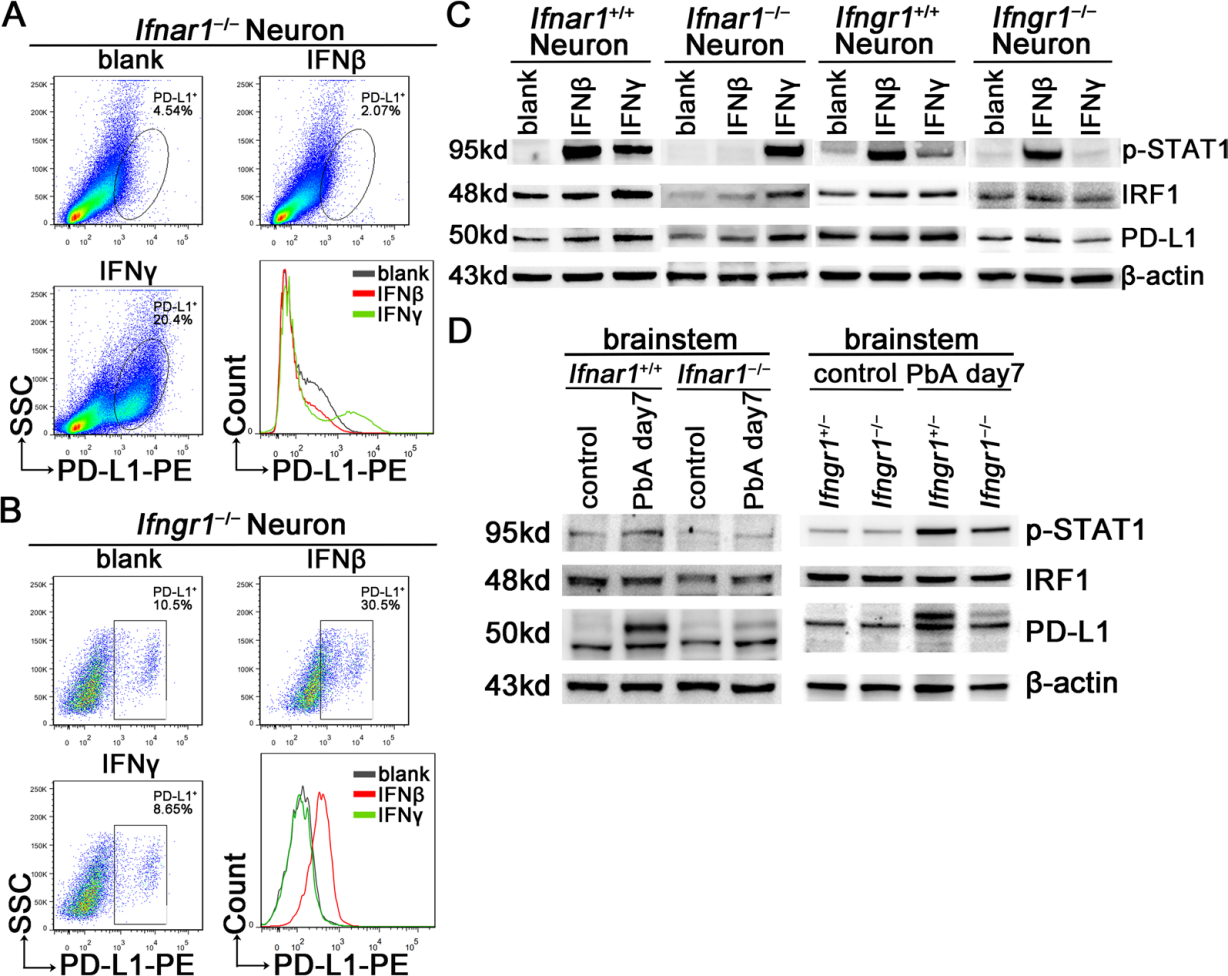

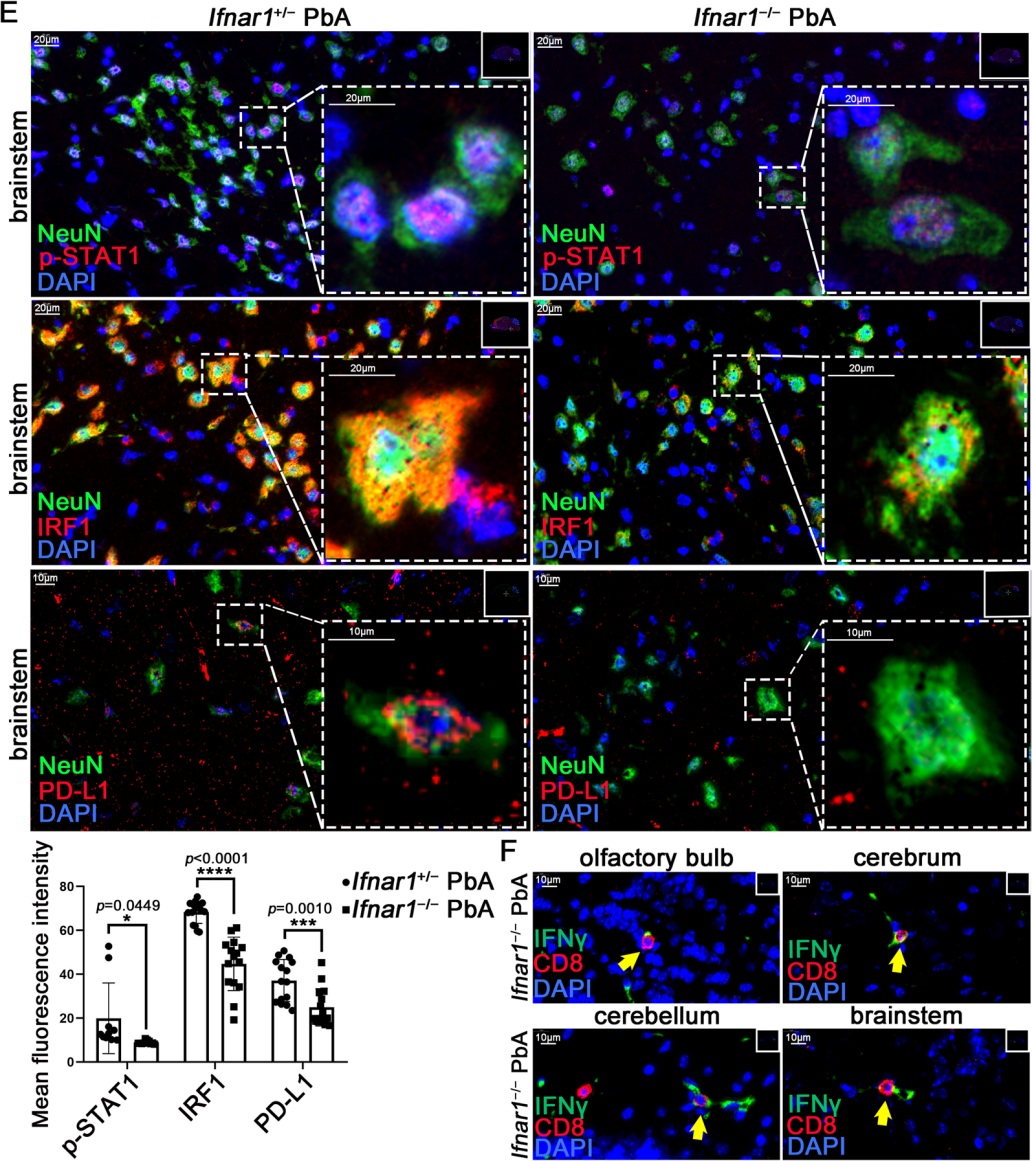
The STAT1/IRF1 pathway in *Ifnar1*^−/−^ or *Ifngr1*^−/−^ mice after PbA infection. **A** Flow cytometry of PD-L1 levels on *Ifnar1*^−/−^ neurons stimulated with IFNβ or IFNγ. **B** Flow cytometry of PD-L1 levels on *Ifngr1*^−/−^ neurons stimulated with IFNβ or IFNγ. **C** WB analysis of p-STAT1, IRF1, and PD-L1 levels in *Ifnar1*^+/+^ or *Ifnar1*^−/−^ neurons, and *Ifngr1*^+/+^ or *Ifngr1*^−/−^ neurons stimulated with IFNβ or IFNγ. **D** WB analysis of p-STAT1, IRF1, and PD-L1 in the brainstem of *Ifnar1*^+/+^ or *Ifnar1*^−/−^ mice, and *Ifngr1*^+/−^ or *Ifngr1*^−/−^ mice with PbA infection, uninfected littermates as control. **E** IF staining of p-STAT1, IRF1, and PD-L1 in neurons (NeuN^+^) in the brainstem of *Ifnar1*^+/−^ and *Ifnar1*^−/−^ mice with PbA infection. The mean fluorescence intensity is expressed as mean ± SD; unpaired t-test; n ≥ 10 per group. **F** IF staining of the infiltrating IFNγ^+^CD8^+^ T cells (yellow arrow) in the olfactory bulb, cerebrum, cerebellum, and brainstem of *Ifnar1*^−/−^ mice with PbA infection

The primary cortical neurons of mice genetically lacking IFNAR or IFNGR were separately cultured to further investigate the crucial role of the IFN pathway in neuronal PD-L1 expression. The PD-L1 levels in neurons were detected using FC. The levels of PD-L1 in *Ifnar1*^−/−^ neurons did not increase with IFNβ stimulation but significantly increased with IFNγ stimulation (Fig. 5A and S5A), while in *Ifngr1*^−/−^ neurons, the PD-L1 levels did not increase with IFNγ stimulation (Fig. 5B and S5B). In addition, compared with *Ifngr1*^+/−^ neurons, the increased PD-L1 levels in *Ifngr1*^−/−^ neurons induced by ECM CD8^+^ T cells were weakened (Figure S5B). WB analysis of p-STAT1, IRF1, and PD-L1 showed the same changes as FC when *Ifnar1*^−/−^ or *Ifngr1*^−/−^ neurons were stimulated with IFNβ or IFNγ (Fig. 5C and S5C).

*Ifnar1*^−/−^ and *Ifngr1*^−/−^ mice were separately infected with PbA, and WB analysis of the mouse brainstems showed that the levels of p-STAT1 and PD-L1 in knockout mice increased to a certain extent after infection but were lower than those of the control littermates (Fig. 5D and S5D). IF staining of mouse brains at day 7 post-infection showed that the levels of p-STAT1, IRF1, and PD-L1 in *Ifnar1*^−/−^ and *Ifngr1*^−/−^ mice were lower than those in control littermates (Figures S5E and S5F). NeuN IF staining revealed that the expression of p-STAT1, IRF1, and PD-L1 in neurons of *Ifnar1*^−/−^ mice infected with PbA was significantly reduced compared to that of the *Ifnar1*^+/−^ mice infected with PbA (Fig. 5E). Similar changes were observed in the *Ifngr1*^−/−^ mice (Figures S5G, S5H, and S5I). Further studies showed that numerous infiltrating CD8^+^ T cells were present in the brains of *Ifnar1*^−/−^ or *Ifngr1*^−/−^ mice infected with PbA (Fig. 5F and S5J), and CD8^+^ T cells in the brainstem secreted IFNγ (Fig. 5F and S5K), implying that IFNAR or IFNGR deficiency did not affect the inflammatory function of infiltrating CD8^+^ T cells.

### The protective effect induced by enhancing the PD-L1 expression in neuron or ECM brain

**Fig. 6:**
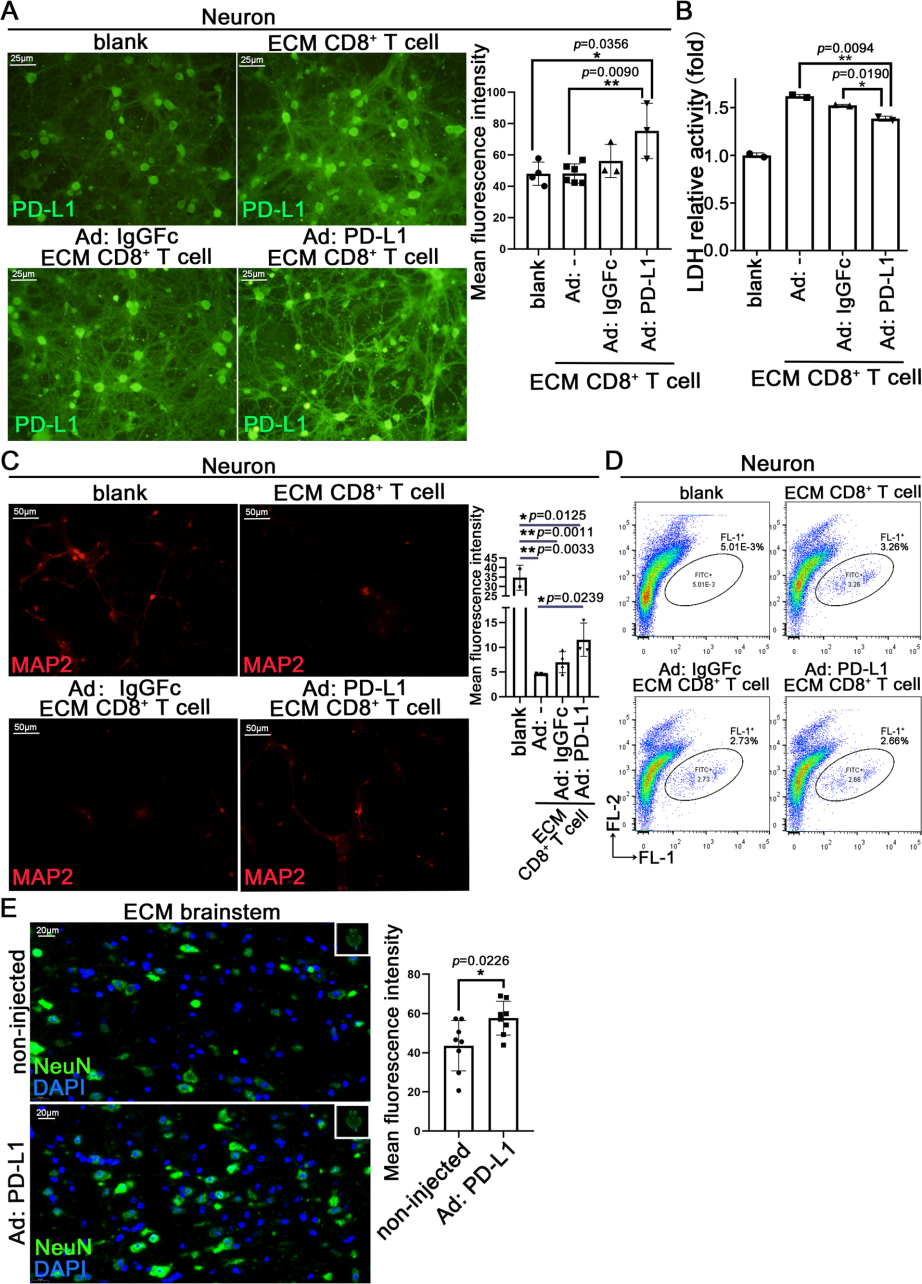

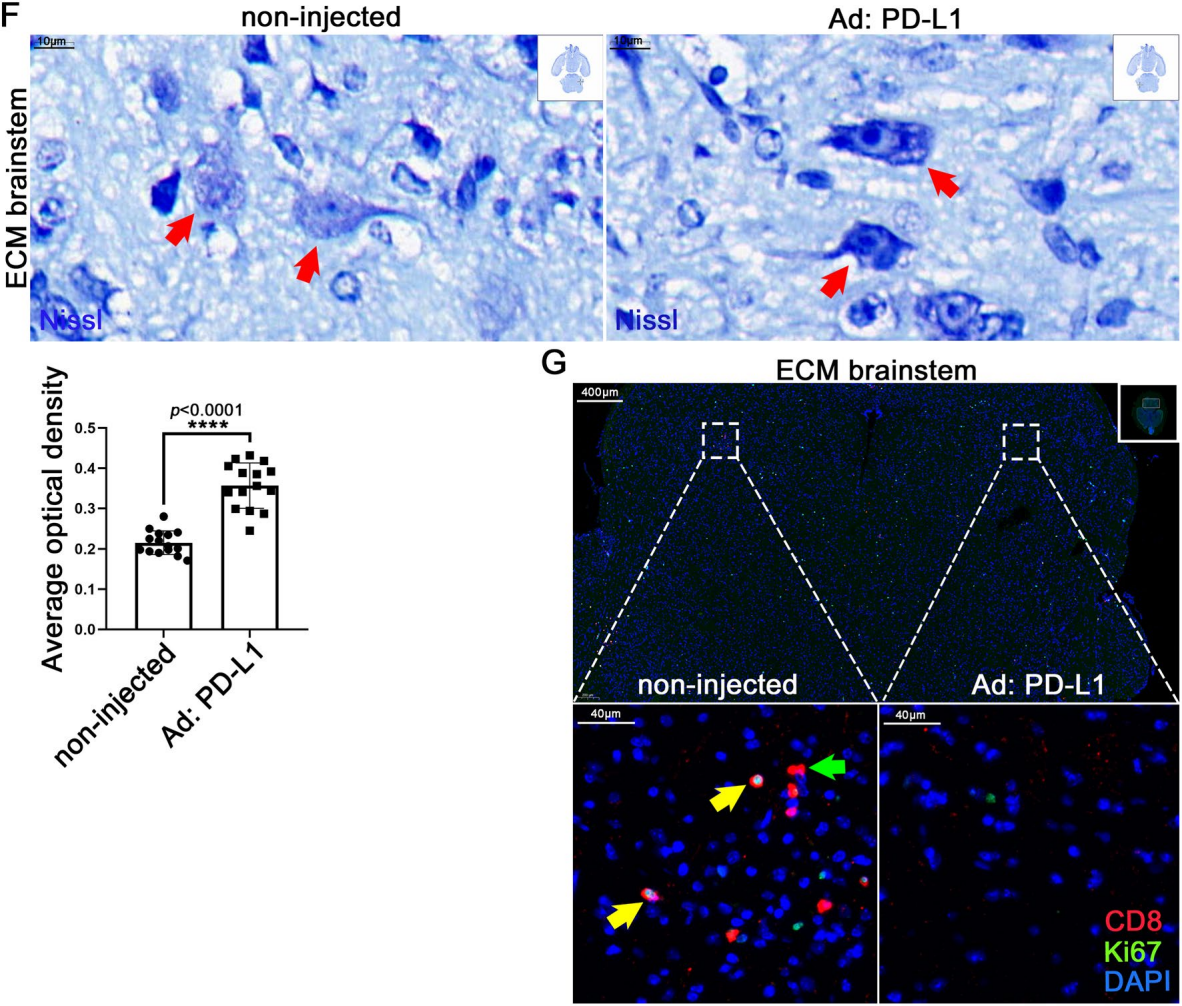
The protective effects of PD-L1 expression vector in neurons and ECM brains. **A** IF staining of PD-L1 in neurons co-cultured with ECM CD8^+^ T cells, after treatment with recombinant adenovirus expressing PD-L1 IgGFc fusion protein (Ad: PD-L1) or recombinant adenovirus expressing IgGFc (Ad: IgGFc). **B** LDH activity in supernatants of Ad: PD-L1 or Ad: IgGFc treated neurons co-cultured with ECM CD8^+^ T cells. Data are expressed as mean ± SD; unpaired t-test, n = 2 per group. **C** IF staining of MAP2 in Ad: PD-L1 or Ad: IgGFc treated neurons co-cultured with ECM CD8^+^ T cells. **D** Flow cytometry for JC-1 (FL-1: monomer, FL-2: aggregates) in Ad: PD-L1 or Ad: IgGFc treated neurons co-cultured with ECM CD8^+^ T cells. **E** IF staining of NeuN in neurons on Ad: PD-L1 injected side or non-injected side of ECM brainstem. **A**–**E** Fluorescence statistical data are expressed as mean ± SD; unpaired t-test; n ≥ 3 per group. F) Nissl staining of neurons (red arrow) on Ad: PD-L1 injected side or non-injected side of ECM brainstem. Data are expressed as mean ± SD; unpaired t-test; n = 15 fields per group. **G** IF staining of Ki67 and CD8 on Ad: PD-L1 injected side or non-injected side of ECM brainstem (yellow arrow: Ki67^+^CD8^+^ T cell; green arrow: CD8^+^ T cell)

We exposed neurons overexpressing PD-L1 to ECM CD8^+^ T cells to verify the protective effect of PD-L1 on neurons. Neurons treated with recombinant adenovirus expressing PD-L1 IgGFc fusion protein (Ad: PD-L1) (Fig. 6A) reduced the damaging effect of ECM CD8^+^ T cells to a certain extent (Figure S6A), and the relative activity of LDH also significantly decreased in the co-culture supernatant (Fig. 6B). IF staining of MAP2 showed that the Ad: PD-L1 could partially protect neurons damaged by CD8^+^ T cells (Fig. 6C), consistent with NeuN IF staining results (Figure S6B). JC-1 staining showed that when stimulated with ECM CD8^+^ T cells, the monomer of JC-1 in the mitochondria of Ad: PD-L1 treated neurons decreased compared with untreated neurons (Fig. 6D). However, no significant difference was observed in the number of cell death using FC of PI staining compared with that of the IgGFc control (Figure S6C).

The Ad: PD-L1 was injected into the brainstem of mice infected with PbA using the stereotactic microinjection technique; the injected area was in the brainstem’s left hemisphere only, the right hemisphere as control (Figure S6D). H&E staining of the non-injected side of the brainstem showed significant hemorrhagic spots, whereas no such spots were observed on the injected side (Figure S6E). IF staining showed increased PD-L1 levels (Figure S6F) on the injected side of brainstem, and more NeuN staining signals on the injected side indicated a higher number of neurons (Fig. 6E). Compared with the non-injected side, the Nissl bodies of the neurons on the injected side increased significantly (Fig. 6F), suggesting an improved neural function. Notably, several infiltrating CD8^+^ T cells were observed on the non-injected side of the brainstem, some of which were proliferative, but no CD8^+^ T cells were observed on the injected side (Fig. 6G).

## Discussion

Combined with postmortem and mechanism analysis on ECM mice, neuroinflammation after BBB disruption leads to axonal damage and neurodegeneration, resulting in reversible or irreversible neurological impairment and sequelae in patients CM [36, 37]. As a direct driver of neuronal damage and inflammatory and degenerative CNS diseases, the critical role of CD8^+^ T cells in CM development is increasingly recognized [38, 39]. However, few evidence has been reported on the interaction between infiltrating CD8^+^ T cells and neurons. Our previous research has shown that ECM CD8^+^ T cells induce neuronal ferroptosis [29], and this study demonstrates that CD8^+^ T cells are associated with nerve damage in various cell death pathways during ECM, including autophagy and apoptosis. In the co-culture system, ECM-activated CD8^+^ T cells contacted and destroyed the axons, confirming that CD8^+^ T cells directly participate in neuronal injury, leading to neurological sequelae.

In the pathological process of CM, IFNγ is the main driver and is mostly secreted by CD8^+^ T cells within the cerebral micro-vessels during ECM [40, 41]. Therefore, we suggest that infiltrating CD8^+^ T cells are the main source of IFNγ in the brain parenchyma during ECM. The type I IFNs play a more complex role during malaria, such as promoting parasite clearance in the liver stage, suppressing IFNγ production of parasitic-specific CD4^+^ T cells, and promoting IL-10 producing Th1 cells [42, 43]. However, the source of IFNβ in the CNS needs to be further determined and we speculated that type I IFNs in peripheral blood infiltrated into the brain parenchyma after BBB destruction or may be secreted by specific nerve cells.

In addition to immune activation, the immunomodulatory functions of IFN, particularly in regulating PD-L1 expression in cancer and inflammatory diseases, have been reported [44–47]. In autoimmune CNS disease, neurons inhibit T cell function by promoting T cells transformation into T regulatory cells or by inducing the apoptosis of T cells via Fas ligand [48, 49]. However, the direct inhibition of T cells by neurons is rarely observed. This study is the first to report the immunomodulatory effects of neurons in the brain of an ECM model. scRNA-seq implied various types of nerve cells, including neurons, upregulated the immune checkpoint molecule PD-L1 levels in ECM mice. However, the number of neurons detected through scRNA-seq was relatively small due to the cell separation methods. We also demonstrated that neurons of ECM mice in vitro and neurons stimulated by IFNs or ECM-activated CD8^+^ T cells in vitro upregulated PD-L1 expression, and the upregulated PD-L1 expression protected neurons themselves.

The IFNγ/p-STAT pathway plays an important role in the CNS immunomodulatory function. The CD8^+^ T cells promoted the differentiation of neural stem cells into astrocytes by the IFNγ/STAT1/GFAP axis [50], and STAT1 and STAT3 have been reported as target genes for the upregulation of PD-L1 in STAT family members induced by inflammatory cytokines such as IFNγ [51]. However, studies on tumor immunity have also found that the knockout of STAT1 and STAT3 does not affect the constitutive expression of PD-L1 [52], indicating a variety of cytokines may induce the expression of PD-L1 on tumor and/or immune cells through different signaling mechanisms. In addition, there is no clear evidence to support the induction of PD-L1 expression by IFNβ via STAT pathway. We confirmed that IFNβ and IFNγ could induce neuronal PD-L1 expression through the STAT1/IRF1 pathway, which plays a crucial pro-inflammatory role in the peripheral immune system by rapidly activating and deactivating to prevent excessive inflammation [53]. The expression of p-STAT1, IRF1, and PD-L1 in neurons was upregulated in the brains of ECM mice, and the absence of the IFN receptor resulted in lower levels of neuroinflammation and less nerve cell damage. Cross-regulation between type I and type II IFNs has been reported, and there may also be synergistic or competitive effects between the two IFN pathways [43, 54–56]. However, the effects of the IFN/STAT1 immune signaling pathway on neuronal homeostasis and pathology remain unclear.

CD8^+^ T cells directly target cells through an immunological synapse comprising the T-cell receptor, MHC-I molecules, and adhesion molecules, resulting in target cell damage [39, 57–59]. We observed a significant upregulation of MHC-I molecule expression in neurons after being stimulated by CD8^+^ T cells, implying that neurons “internalize” *Plasmodium* antigens, which are still unknown during ECM, and present them on the cell surface as peptide-MHC-I complex, which could be recognized by CD8^+^ T cells. As MHC-I plays a complex role in the CNS, it may exacerbate brain injury after ischemia [60] and limit synaptic plasticity in healthy neurons [61], suggesting that the high expression of neuronal MHC-I molecules is associated with ECM sequelae. In the mouse model of sciatic nerve injury, the dorsal root ganglia sensory neurons express chemokine CXCL13 to attract CXR5^+^CD8^+^ T cells and act as antigen-presenting cells by overexpressing MHC-I to activate Caspase 3/p-AKT/pS6 signaling in T cells, leading to regenerative failure [62]. The neuroantigen-specific CD8^+^ T cells infiltrate and injure antigen-presenting axons driving by demyelination in multiple sclerosis (MS) [63]. The presentation of MHC-I molecules in dopaminergic neurons is indeed accompanied by an increase in the number of CD8^+^ T cells [64]. Additionally, in our study some CD8^+^ T cells co-cultured with neurons show proliferation after neuronal contact but die after 3–4 h due to cell enlargement and increased membrane permeability. Consequently, the pathway through which neurons activate T cells and the mode of CD8^+^ T cell death in this process still need further exploration.

The brain stereotaxic injection system can directly deliver drugs to the target areas of brain tissue, which is crucial for researching nervous system diseases, especially in the study of animal models. However, as with other surgical procedures, the greatest difficulty of brain stereotaxic injection surgery is ensuring the survival of the animal. Therefore, a low injection amount is administered and it is difficult to inject multiple times continuously. In particular, brain stereotaxic injection is an invasive operation, which will definitely cause BBB injury. However, it is necessary to further clarify whether this mechanical injury will affect the subsequent neuroinflammation caused by PbA. PD-L1 fusion protein or expression vector is a promising immunoadjuvant therapy for cerebral malaria. Further studies are needed to solve the problem of drugs crossing the BBB and the combination with anti-malarial drugs.

The scRNA-seq analysis of ECM mouse brain stem also showed that microglia and astrocytes, the main resident cells in the brain, showed activation characteristics. Notably, the PD-1/PD-L1 pathway was activated in the microglia, and intrathecal injection of recombinant adenovirus expressing PD-L1 could inhibit neuroinflammation and alleviate ECM symptoms [16]. After PbA infection, numerous astrocytes maintain continuous activation of infiltrating CD8^+^ T cells in ECM brain tissue [14]. Therefore, the appropriate enhancement of the PD-1/PD-L1 pathway during ECM may remold nerve homeostasis, which could target CD8^+^ T cells and influence resident cells in the brain.

## Conclusions

We elucidated the role of ECM-activated CD8^+^ T cells in the pathogenesis of neuronal injury in ECM mice. We observed neuronal damage caused by infiltrating CD8^+^ T cells. More importantly, we reported the immunomodulatory role of neurons in the inflammatory microenvironment of the ECM mouse brain by upregulating PD-L1 through the STAT1/IRF1 pathway induced by type I or type II IFNs to inhibit the cytotoxicity of infiltrating CD8^+^ T cells, thereby alleviating brain immunopathological damage. The immune modulation is a potential approach to prevent or reverse the neuroimmune injury of CM [2, 65], and our study provides a potential direction for this approach. In addition to neurons and infiltrating immune cells, microglia, astrocytes and other nerve cells also participate in the formation of the ECM neuroimmune microenvironment. Their immunomodulatory functions and interactions ultimately determine the extent and outcome of neuroinflammation and nerve injury in ECM progression. Therefore, the study of neuro-immune cell interaction will provide an essential theoretical basis for ameliorating the severity and prognosis of neuroinflammatory diseases besides CM.

### Supplementary Information

Supplementary Material 1: Fig. S1. Nerve cell injury and activated CD8^+^ T cell infiltration in the ECM mouse brain. A) H&E staining of ECM brains showed multiple spots of intracerebral hemorrhage (dark blue arrow). B) IHC staining of synaptophysin (light blue arrow) in the cerebrum of control and ECM mice. C) Nissl staining of neurons (pink arrow) in the brainstem of control and ECM mice. Data are expressed as mean ± SD; n = 8 fields per group. D) IF staining of TUNEL^+^ cells in the olfactory bulb, cerebrum, cerebellum, and brainstem of control and ECM mice. E) IF staining of LC3 in neurons in the cerebrum of control and ECM mice. F) IF staining of Ki67^+^CD8^+^ T cells (yellow arrow) in the olfactory bulb of ECM mice. Fig. S2 The interaction of neurons and ECM CD8^+^ T cells in vitro. A) IF staining of naïve or ECM CD8^+^ T cells (yellow arrow) adhering to neurons (left image) and quantification of adhered CD8^+^ T cells (right image). Data are expressed as mean ± SD; unpaired t-test, n > 3 sections per group. B) CCK-8 detection in the supernatant of neurons treated with different proportions of CD8^+^ T cell culture supernatant. Data are expressed as mean ± SD; unpaired t-test, n = 4 per group. C) Flow cytometry of JC-1 (FL-1: monomer, FL-2: J-aggregates) in neurons co-cultured with ECM CD8^+^ T cell. D) IF staining of ECM CD8^+^ T cell (yellow arrow) adhering to axon. E) q-PCR detection of the *H2-D1* expression in neurons co-cultured with ECM CD8^+^ T cell. Data are expressed as mean ± SD; unpaired t-test, n = 3 per group. F) Flow cytometry of the H2-D/K levels on neurons co-cultured with ECM CD8^+^ T cell. G) IF staining of H2-D/K and CD18 in CD8^+^ T cell (yellow arrow) and neuron (white arrow) co-culture system. Fig. S3 IFNβ or IFNγ induces neurons to upregulate PD-L1. A) IHC staining of PD-L1 in the olfactory bulb, cerebrum, and cerebellum of control and ECM mice (red arrow: PD-L1^+^ nerve cells). B) q-PCR analysis of *Cd274* expression in neurons with IFNβ (100 U/mL, the same below) or IFNγ (20 ng/mL, the same below) stimulation at different points in time. Data are expressed as mean ± SD; unpaired t-test, n = 3 per group. C) WB analysis of PD-L1 levels in neurons with IFNβ stimulation at different points in time. D) IF staining of PD-L1 in neurons stimulated with IFNβ or IFNγ. Fig. S4 IFNβ or IFNγ induces the expression of PD-L1 through the STAT1/IRF1 pathway in neurons. RNA-seq was performed on neurons stimulated with IFNγ (20 ng/mL, the same below) or ECM CD8^+^ T cells for 24 h. A) The Gene Ontology enrichment dot plot of IFN-related pathways in neurons stimulated with ECM CD8^+^ T cells. B) Violin plots of *Cd274*, *Stat1*, and *Irf1* expression in neurons with IFNγ or ECM CD8^+^ T cell stimulation (*P*_adj_ < 0.05). C) q-PCR detection of *Stat1* and *Irf1* expression in neurons stimulated at different times with IFNβ (100 U/mL, the same below) or IFNγ. Data are expressed as mean ± SD; unpaired t-test, n = 3 per group, ****P < 0.0001. D) WB analysis of p-STAT1 in neurons stimulated at different times with IFNβ or IFNγ. E) IF staining of p-STAT1 in neurons with IFNβ or IFNγ stimulation. F) q-PCR detection of *Cd274*, *Stat1,* and *Irf1* in neurons treated with fludarabine (20 µM), under IFNβ or IFNγ stimulation. Data are expressed as mean ± SD; unpaired t-test, n = 3 per group. G) Flow cytometry of PD-L1 on neurons treated with fludarabine, IFNβ or IFNγ. H) WB analysis of p-STAT1, IRF1, and PD-L1 in neurons treated with IFNγ and different doses of fludarabine. I) IHC staining of IRF1 in the olfactory bulb, cerebrum, and cerebellum of control and ECM mice. Fig. S5 Changes of the *Ifnar1*^−/−^ or *Ifngr1*^−/−^ neurons in ECM brain or in vitro treated with IFNs. A) Flow cytometry of PD-L1 on *Ifnar1*^+/−^ and *Ifnar1*^−/−^ neurons treated with IFNβ or IFNγ. B) Flow cytometry of PD-L1 on *Ifngr1*^+/−^ and *Ifngr1*^−/−^ neurons treated with IFNγ or ECM CD8^+^ T cells. C) Statistical results of WB analysis of p-STAT1, IRF1, and PD-L1 levels in *Ifnar1*^+/+^ or *Ifnar1*^−/−^, and *Ifngr1*^+/+^ or *Ifngr1*^−/−^ neurons treated with IFNβ or IFNγ. Data are mean ± SD; unpaired t-test, n ≥ 3 per group. D) Statistical results of WB analysis of p-STAT1, IRF1, and PD-L1 levels in the brainstem of *Ifnar1*^+/+^ or *Ifnar1*^−/−^, and *Ifngr1*^+/−^ or *Ifngr1*^−/−^ mice with PbA infection, uninfected littermates as control. Data are expressed as mean ± SD; unpaired t-test, n ≥ 3 per group. E) IF staining of p-STAT1, IRF1, and PD-L1 in the brainstem of *Ifnar1*^+/+^ and *Ifnar1*^−/−^ mice infected with PbA (yellow arrow: positive cell). F) IF staining of p-STAT1, IRF1, and PD-L1 in the brainstem of *Ifngr1*^+/+^ and *Ifngr1*^−/−^ mice infected with PbA (yellow arrow: positive cell). G-I) IF staining of PD-L1, p-STAT1, and IRF1 in the brainstem of *Ifngr1*^+/+^ and *Ifngr1*^−/−^ mice infected with PbA, neurons tagged with NeuN (yellow arrow: neuron). J) IF staining of the infiltrating CD8^+^ T cells (yellow arrow) in the brains of *Ifngr1*^−/−^ mice infected with PbA. K) IF staining of the infiltrating IFNγ^+^CD8^+^ T cells (yellow arrow) in the brainstem of *Ifngr1*^−/−^ mice infected with PbA. Fig. S6 Upregulation of PDL1 protects neurons in vitro and in ECM brain. A) Optical microscope images of the Ad: PD-L1 or Ad: IgGFc treated neurons stimulated with ECM CD8^+^ T cells. B) IF staining of NeuN in Ad: PD-L1 or Ad: IgGFc treated neurons stimulated with ECM CD8^+^ T cells. C) Flow cytometry of PI^+^ neurons in Ad: PD-L1 or Ad: IgGFc treated neurons stimulated with ECM CD8^+^ T cells. D) The diagram of stereotaxic microinjection of the brainstem in mice (red dot: injection location). E) H&E staining on the Ad: PD-L1 injected side or non-injected side of ECM brainstem (yellow arrow: hemorrhage). F) IF staining of PD-L1 on the Ad: PD-L1 injected side or non-injected side of ECM brainstem.

Supplementary Material 2: Table S1. List of primer sequences used for q-PCR.

Supplementary Material 3: Video 1. Live-cell time-lapse photography detection showed ECM CD8^+^ T cells causing the death of primary cultured cortical neurons (every 30 s for a total of 4 h).

Supplementary Material 4: Video 2. ECM CD8^+^ T cells adhering to and damaging the exons of neurons in time-lapse photography.

Supplementary Material 5: Video 3. Time-lapse photography showed ECM CD8^+^ T cell proliferation after interacting with neurons.

## Data Availability

Data sets generated and analyzed during the current study are available from the corresponding author upon reasonable request. Our scRNA-seq data under the BioProject ID: PRJNA735877 have been submitted to the database of the NCBI Sequence Read Archive (http://www.ncbi.nlm.nih.gov/bioproject/).

## Abbreviations

APP: Amyloid-β precursor protein
BBB: Blood–brain barrier
CNS: Central nervous system
CM: Cerebral malaria
DAB: Diaminobenzidine
DMSO: Dimethyl sulfoxide
ECM: Experimental cerebral malaria
FACS: Fluorescence-activated cell sorting
FBS: Fetal bovine serum
IF: Immunofluorescence
IHC: Immunohistochemistry
IFN: Interferon
LDH: Lactate dehydrogenase
PCR: Polymerase chain reaction
PI: Propidium iodide
RBC: Red blood cells
TUNEL: Terminal deoxynucleotidyl transferase dUTP nick-end labeling
WB: Western blot
FC: Flow cytometry
q-PCR: Quantitative real-time polymerase chain reaction
