# Supplementary figures and images for: Neurons upregulate PD-L1 via IFN/STAT1/IRF1 to alleviate damage by CD8+ T cells in cerebral malaria
Source: J Neuroinflammation. 2024 May 7;21:119. doi: 10.1186/s12974-024-03114-7 (PMC11077882; doi:10.1186/s12974-024-03114-7)

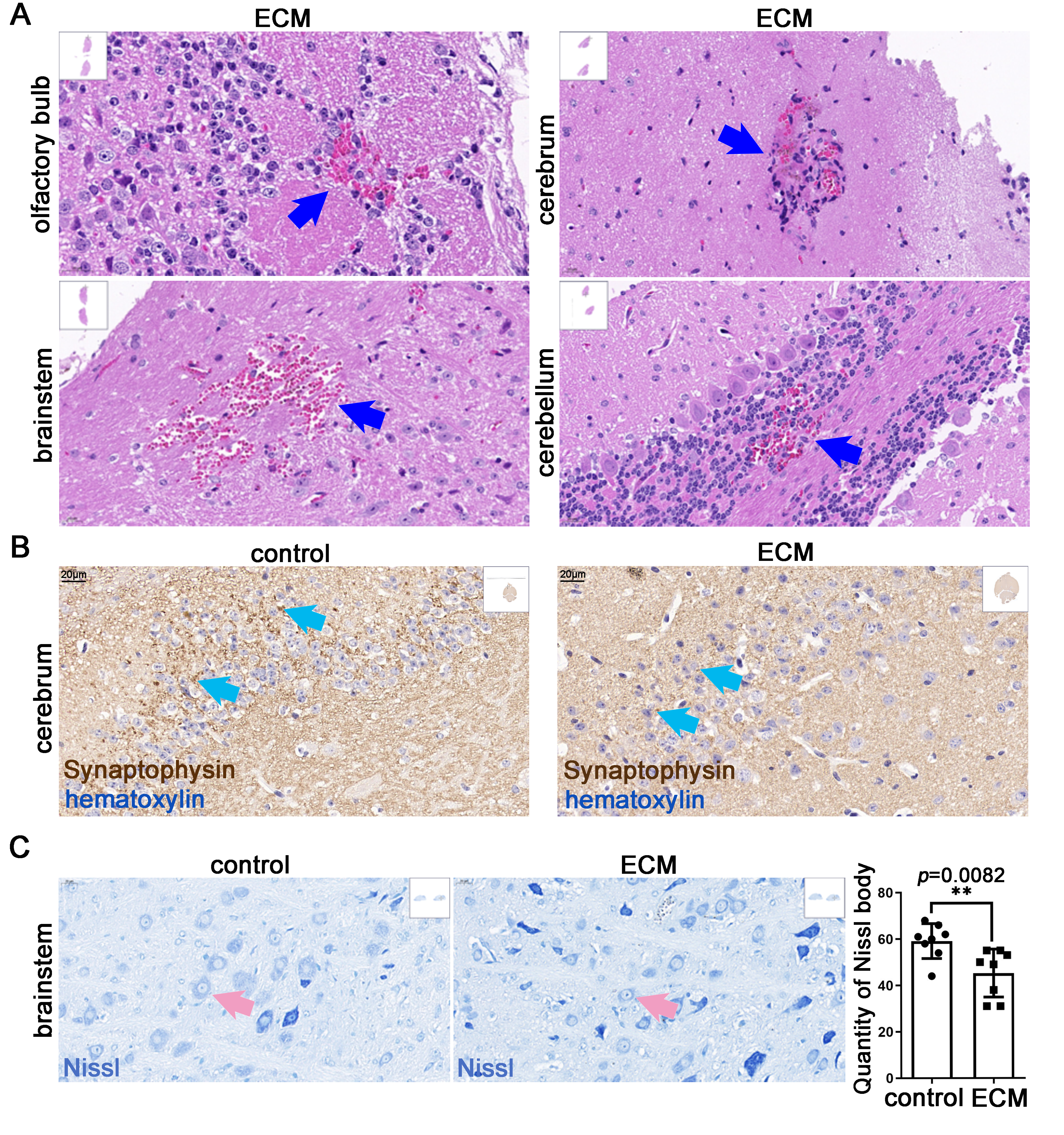

Supplement: Supplementary file 1 — Supplementary Material 1: Fig. S1. Nerve cell injury and activated CD8+ T cell infiltration in the ECM mouse brain. A) H&E staining of ECM brains showed multiple spots of intracerebral hemorrhage (dark blue arrow). B) IHC staining of synaptophysin (light blue arrow) in the cerebrum of control and ECM mice. C) Nissl staining of neurons (pink arrow) in the brainstem of control and ECM mice. Data are expressed as mean ± SD; n = 8 fields per group. D) IF staining of TUNEL+ cells in the olfactory bulb, cerebrum, cerebellum, and brainstem of control and ECM mice. E) IF staining of LC3 in neurons in the cerebrum of control and ECM mice. F) IF staining of Ki67+CD8+ T cells (yellow arrow) in the olfactory bulb of ECM mice. Fig. S2 The interaction of neurons and ECM CD8+ T cells in vitro. A) IF staining of naïve or ECM CD8+ T cells (yellow arrow) adhering to neurons (left image) and quantification of adhered CD8+ T cells (right image). Data are expressed as mean ± SD; unpaired t-test, n > 3 sections per group. B) CCK-8 detection in the supernatant of neurons treated with different proportions of CD8+ T cell culture supernatant. Data are expressed as mean ± SD; unpaired t-test, n = 4 per group. C) Flow cytometry of JC-1 (FL-1: monomer, FL-2: J-aggregates) in neurons co-cultured with ECM CD8+ T cell. D) IF staining of ECM CD8+ T cell (yellow arrow) adhering to axon. E) q-PCR detection of the H2-D1 expression in neurons co-cultured with ECM CD8+ T cell. Data are expressed as mean ± SD; unpaired t-test, n = 3 per group. F) Flow cytometry of the H2-D/K levels on neurons co-cultured with ECM CD8+ T cell. G) IF staining of H2-D/K and CD18 in CD8+ T cell (yellow arrow) and neuron (white arrow) co-culture system. Fig. S3 IFNβ or IFNγ induces neurons to upregulate PD-L1. A) IHC staining of PD-L1 in the olfactory bulb, cerebrum, and cerebellum of control and ECM mice (red arrow: PD-L1+ nerve cells). B) q-PCR analysis of Cd274 expression in neurons with IFNβ (100 U/mL, the same below) or [file 12974_2024_3114_MOESM1_ESM.zip › Supplementary figure1-6/Figure S1 A-C ╨í╫╓║┼.png]

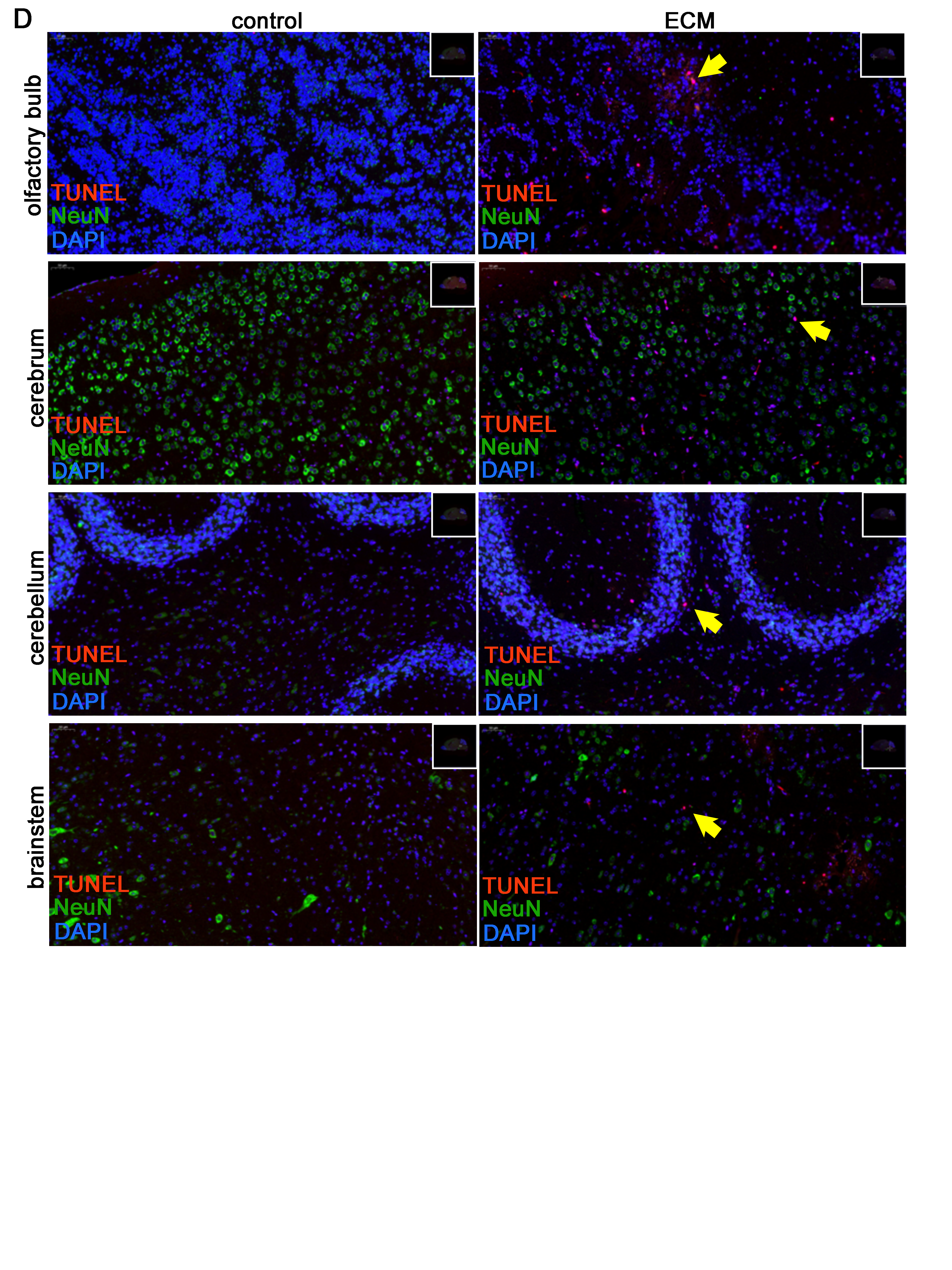

Supplement: Supplementary file 1 — Supplementary Material 1: Fig. S1. Nerve cell injury and activated CD8+ T cell infiltration in the ECM mouse brain. A) H&E staining of ECM brains showed multiple spots of intracerebral hemorrhage (dark blue arrow). B) IHC staining of synaptophysin (light blue arrow) in the cerebrum of control and ECM mice. C) Nissl staining of neurons (pink arrow) in the brainstem of control and ECM mice. Data are expressed as mean ± SD; n = 8 fields per group. D) IF staining of TUNEL+ cells in the olfactory bulb, cerebrum, cerebellum, and brainstem of control and ECM mice. E) IF staining of LC3 in neurons in the cerebrum of control and ECM mice. F) IF staining of Ki67+CD8+ T cells (yellow arrow) in the olfactory bulb of ECM mice. Fig. S2 The interaction of neurons and ECM CD8+ T cells in vitro. A) IF staining of naïve or ECM CD8+ T cells (yellow arrow) adhering to neurons (left image) and quantification of adhered CD8+ T cells (right image). Data are expressed as mean ± SD; unpaired t-test, n > 3 sections per group. B) CCK-8 detection in the supernatant of neurons treated with different proportions of CD8+ T cell culture supernatant. Data are expressed as mean ± SD; unpaired t-test, n = 4 per group. C) Flow cytometry of JC-1 (FL-1: monomer, FL-2: J-aggregates) in neurons co-cultured with ECM CD8+ T cell. D) IF staining of ECM CD8+ T cell (yellow arrow) adhering to axon. E) q-PCR detection of the H2-D1 expression in neurons co-cultured with ECM CD8+ T cell. Data are expressed as mean ± SD; unpaired t-test, n = 3 per group. F) Flow cytometry of the H2-D/K levels on neurons co-cultured with ECM CD8+ T cell. G) IF staining of H2-D/K and CD18 in CD8+ T cell (yellow arrow) and neuron (white arrow) co-culture system. Fig. S3 IFNβ or IFNγ induces neurons to upregulate PD-L1. A) IHC staining of PD-L1 in the olfactory bulb, cerebrum, and cerebellum of control and ECM mice (red arrow: PD-L1+ nerve cells). B) q-PCR analysis of Cd274 expression in neurons with IFNβ (100 U/mL, the same below) or [file 12974_2024_3114_MOESM1_ESM.zip › Supplementary figure1-6/Figure S1 D ╨í╫╓║┼ 3-15╕─.png]

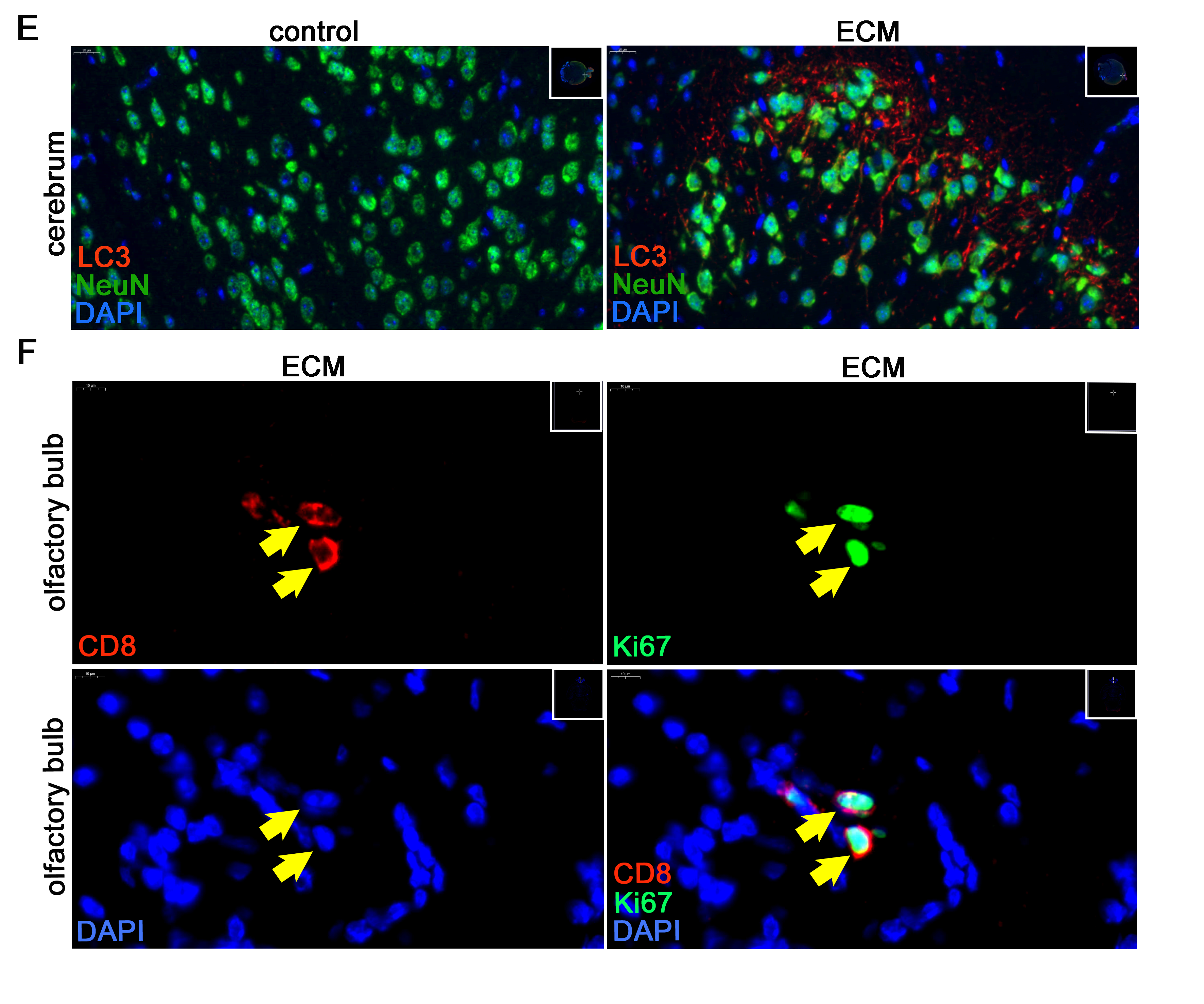

Supplement: Supplementary file 1 — Supplementary Material 1: Fig. S1. Nerve cell injury and activated CD8+ T cell infiltration in the ECM mouse brain. A) H&E staining of ECM brains showed multiple spots of intracerebral hemorrhage (dark blue arrow). B) IHC staining of synaptophysin (light blue arrow) in the cerebrum of control and ECM mice. C) Nissl staining of neurons (pink arrow) in the brainstem of control and ECM mice. Data are expressed as mean ± SD; n = 8 fields per group. D) IF staining of TUNEL+ cells in the olfactory bulb, cerebrum, cerebellum, and brainstem of control and ECM mice. E) IF staining of LC3 in neurons in the cerebrum of control and ECM mice. F) IF staining of Ki67+CD8+ T cells (yellow arrow) in the olfactory bulb of ECM mice. Fig. S2 The interaction of neurons and ECM CD8+ T cells in vitro. A) IF staining of naïve or ECM CD8+ T cells (yellow arrow) adhering to neurons (left image) and quantification of adhered CD8+ T cells (right image). Data are expressed as mean ± SD; unpaired t-test, n > 3 sections per group. B) CCK-8 detection in the supernatant of neurons treated with different proportions of CD8+ T cell culture supernatant. Data are expressed as mean ± SD; unpaired t-test, n = 4 per group. C) Flow cytometry of JC-1 (FL-1: monomer, FL-2: J-aggregates) in neurons co-cultured with ECM CD8+ T cell. D) IF staining of ECM CD8+ T cell (yellow arrow) adhering to axon. E) q-PCR detection of the H2-D1 expression in neurons co-cultured with ECM CD8+ T cell. Data are expressed as mean ± SD; unpaired t-test, n = 3 per group. F) Flow cytometry of the H2-D/K levels on neurons co-cultured with ECM CD8+ T cell. G) IF staining of H2-D/K and CD18 in CD8+ T cell (yellow arrow) and neuron (white arrow) co-culture system. Fig. S3 IFNβ or IFNγ induces neurons to upregulate PD-L1. A) IHC staining of PD-L1 in the olfactory bulb, cerebrum, and cerebellum of control and ECM mice (red arrow: PD-L1+ nerve cells). B) q-PCR analysis of Cd274 expression in neurons with IFNβ (100 U/mL, the same below) or [file 12974_2024_3114_MOESM1_ESM.zip › Supplementary figure1-6/Figure S1 E-F ╨í╫╓║┼.png]

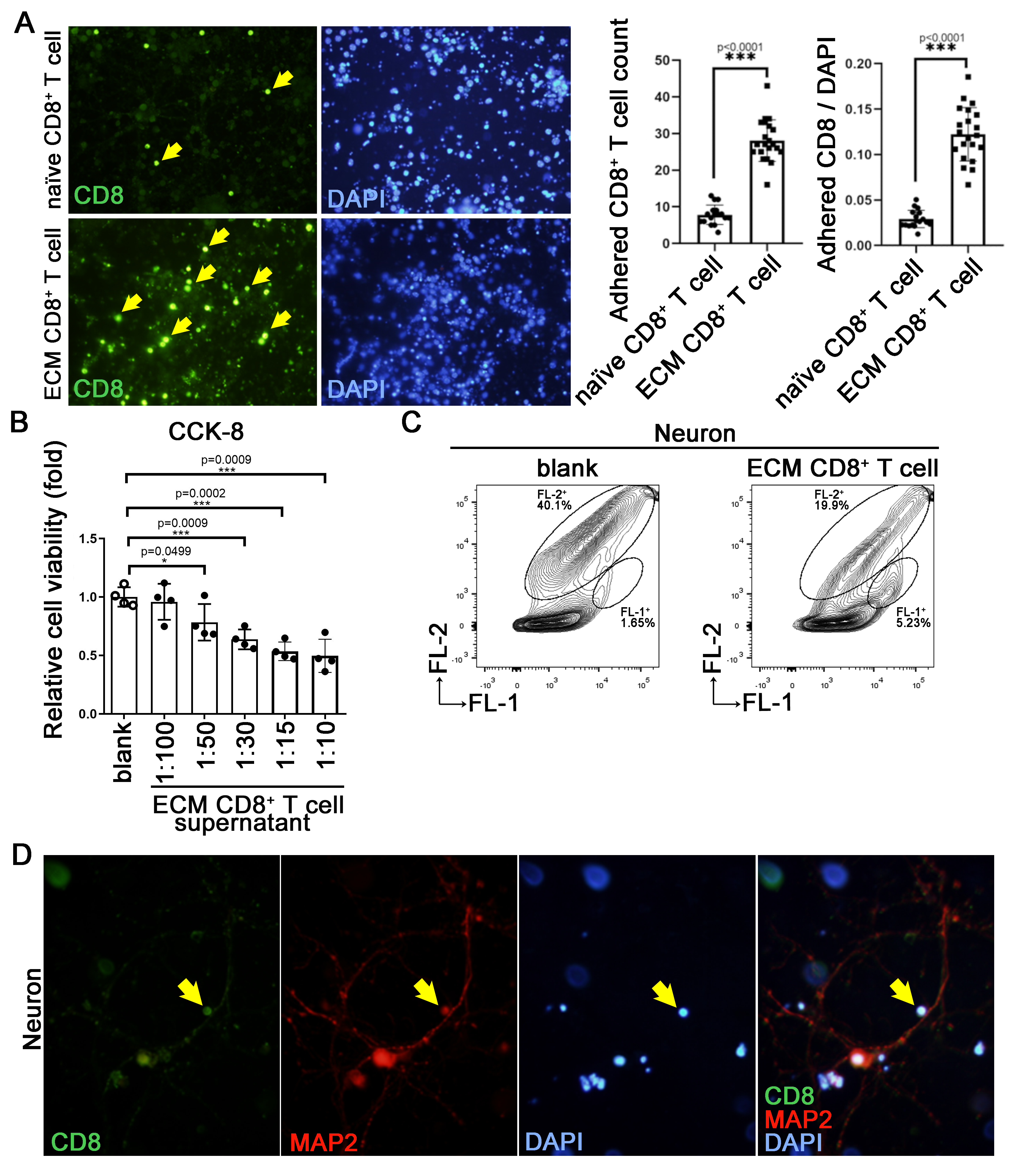

Supplement: Supplementary file 1 — Supplementary Material 1: Fig. S1. Nerve cell injury and activated CD8+ T cell infiltration in the ECM mouse brain. A) H&E staining of ECM brains showed multiple spots of intracerebral hemorrhage (dark blue arrow). B) IHC staining of synaptophysin (light blue arrow) in the cerebrum of control and ECM mice. C) Nissl staining of neurons (pink arrow) in the brainstem of control and ECM mice. Data are expressed as mean ± SD; n = 8 fields per group. D) IF staining of TUNEL+ cells in the olfactory bulb, cerebrum, cerebellum, and brainstem of control and ECM mice. E) IF staining of LC3 in neurons in the cerebrum of control and ECM mice. F) IF staining of Ki67+CD8+ T cells (yellow arrow) in the olfactory bulb of ECM mice. Fig. S2 The interaction of neurons and ECM CD8+ T cells in vitro. A) IF staining of naïve or ECM CD8+ T cells (yellow arrow) adhering to neurons (left image) and quantification of adhered CD8+ T cells (right image). Data are expressed as mean ± SD; unpaired t-test, n > 3 sections per group. B) CCK-8 detection in the supernatant of neurons treated with different proportions of CD8+ T cell culture supernatant. Data are expressed as mean ± SD; unpaired t-test, n = 4 per group. C) Flow cytometry of JC-1 (FL-1: monomer, FL-2: J-aggregates) in neurons co-cultured with ECM CD8+ T cell. D) IF staining of ECM CD8+ T cell (yellow arrow) adhering to axon. E) q-PCR detection of the H2-D1 expression in neurons co-cultured with ECM CD8+ T cell. Data are expressed as mean ± SD; unpaired t-test, n = 3 per group. F) Flow cytometry of the H2-D/K levels on neurons co-cultured with ECM CD8+ T cell. G) IF staining of H2-D/K and CD18 in CD8+ T cell (yellow arrow) and neuron (white arrow) co-culture system. Fig. S3 IFNβ or IFNγ induces neurons to upregulate PD-L1. A) IHC staining of PD-L1 in the olfactory bulb, cerebrum, and cerebellum of control and ECM mice (red arrow: PD-L1+ nerve cells). B) q-PCR analysis of Cd274 expression in neurons with IFNβ (100 U/mL, the same below) or [file 12974_2024_3114_MOESM1_ESM.zip › Supplementary figure1-6/Figure S2 A-D ╨í╫╓║┼.png]

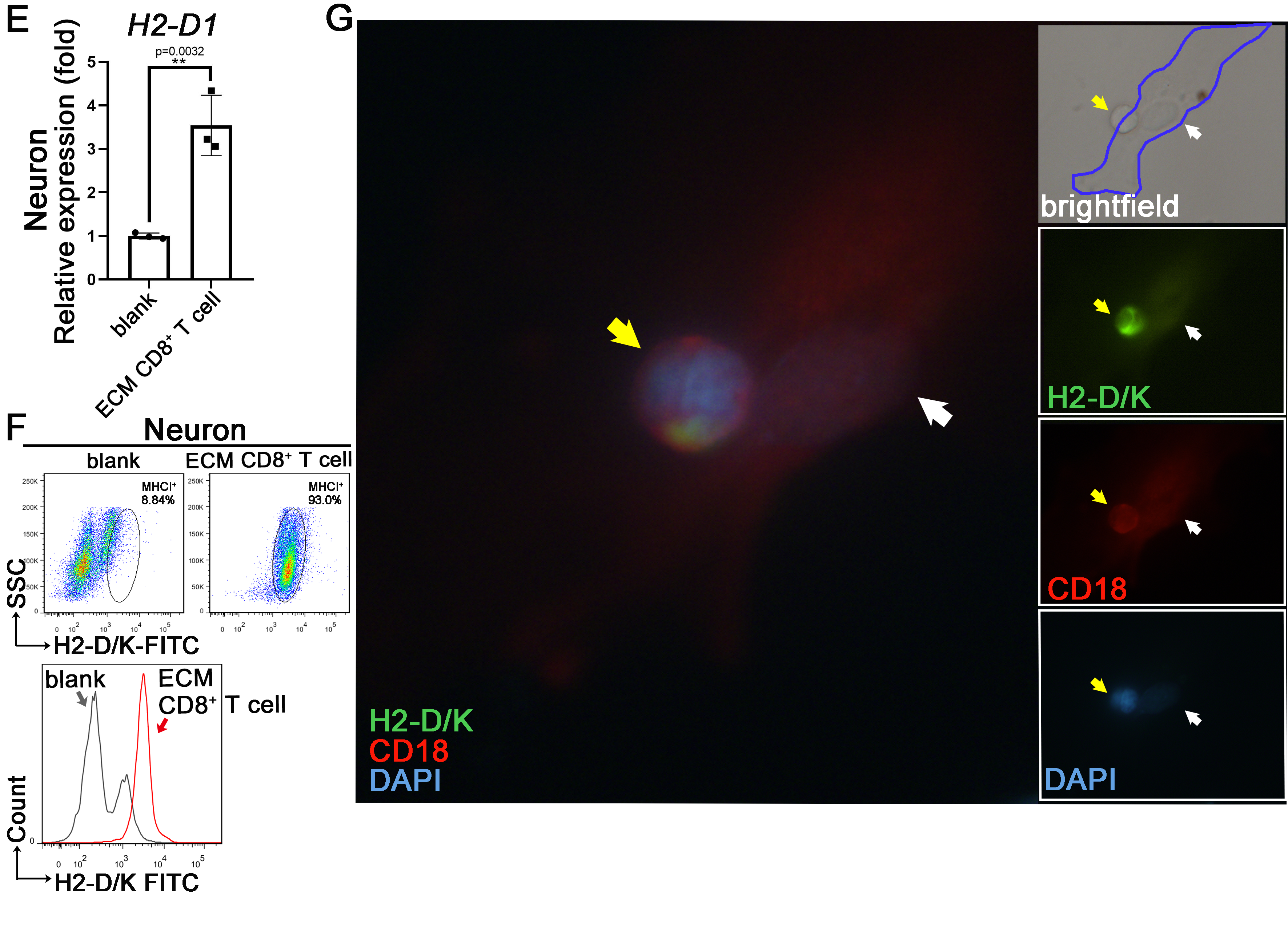

Supplement: Supplementary file 1 — Supplementary Material 1: Fig. S1. Nerve cell injury and activated CD8+ T cell infiltration in the ECM mouse brain. A) H&E staining of ECM brains showed multiple spots of intracerebral hemorrhage (dark blue arrow). B) IHC staining of synaptophysin (light blue arrow) in the cerebrum of control and ECM mice. C) Nissl staining of neurons (pink arrow) in the brainstem of control and ECM mice. Data are expressed as mean ± SD; n = 8 fields per group. D) IF staining of TUNEL+ cells in the olfactory bulb, cerebrum, cerebellum, and brainstem of control and ECM mice. E) IF staining of LC3 in neurons in the cerebrum of control and ECM mice. F) IF staining of Ki67+CD8+ T cells (yellow arrow) in the olfactory bulb of ECM mice. Fig. S2 The interaction of neurons and ECM CD8+ T cells in vitro. A) IF staining of naïve or ECM CD8+ T cells (yellow arrow) adhering to neurons (left image) and quantification of adhered CD8+ T cells (right image). Data are expressed as mean ± SD; unpaired t-test, n > 3 sections per group. B) CCK-8 detection in the supernatant of neurons treated with different proportions of CD8+ T cell culture supernatant. Data are expressed as mean ± SD; unpaired t-test, n = 4 per group. C) Flow cytometry of JC-1 (FL-1: monomer, FL-2: J-aggregates) in neurons co-cultured with ECM CD8+ T cell. D) IF staining of ECM CD8+ T cell (yellow arrow) adhering to axon. E) q-PCR detection of the H2-D1 expression in neurons co-cultured with ECM CD8+ T cell. Data are expressed as mean ± SD; unpaired t-test, n = 3 per group. F) Flow cytometry of the H2-D/K levels on neurons co-cultured with ECM CD8+ T cell. G) IF staining of H2-D/K and CD18 in CD8+ T cell (yellow arrow) and neuron (white arrow) co-culture system. Fig. S3 IFNβ or IFNγ induces neurons to upregulate PD-L1. A) IHC staining of PD-L1 in the olfactory bulb, cerebrum, and cerebellum of control and ECM mice (red arrow: PD-L1+ nerve cells). B) q-PCR analysis of Cd274 expression in neurons with IFNβ (100 U/mL, the same below) or [file 12974_2024_3114_MOESM1_ESM.zip › Supplementary figure1-6/Figure S2 E-G ╨í╫╓║┼.png]

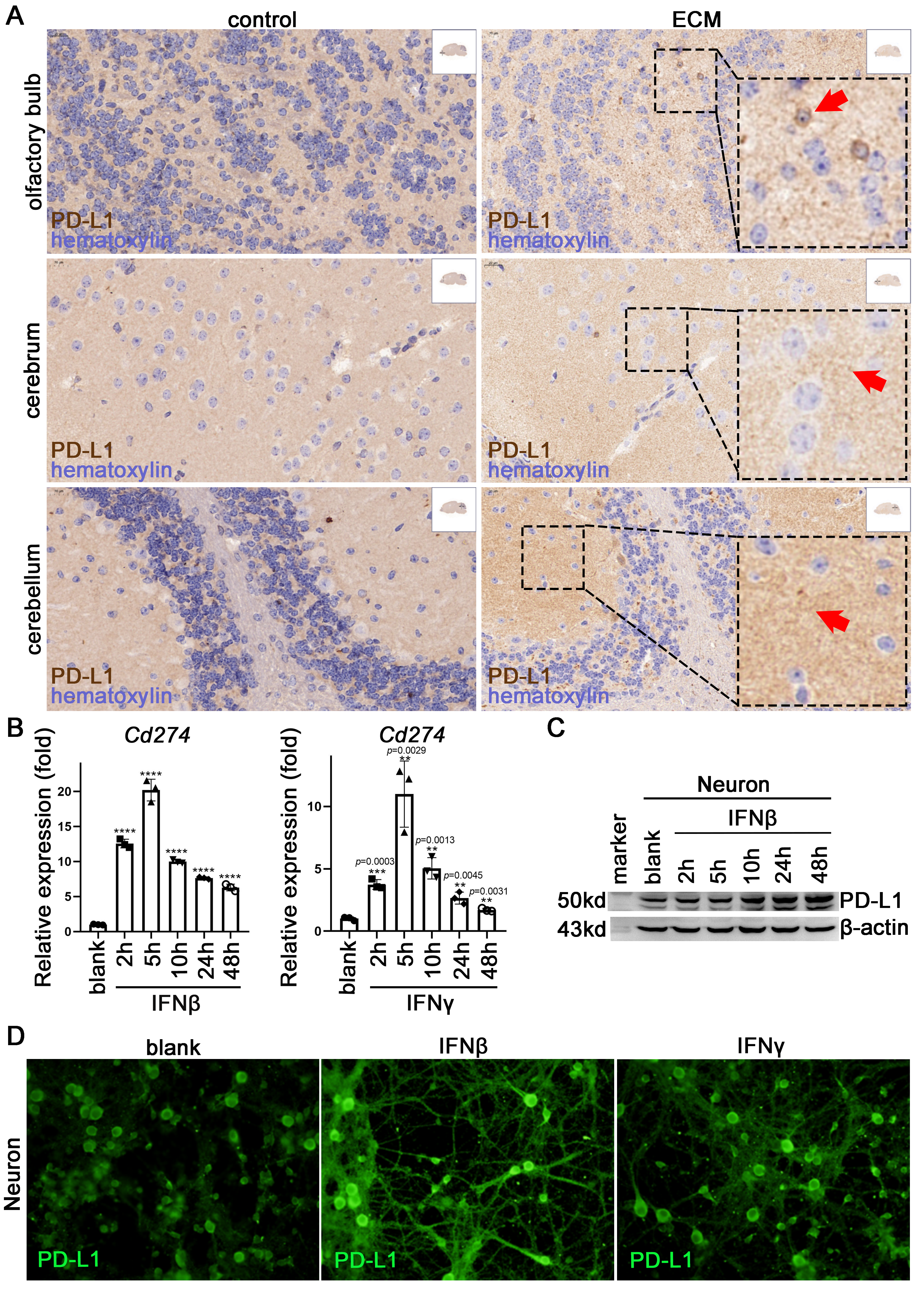

Supplement: Supplementary file 1 — Supplementary Material 1: Fig. S1. Nerve cell injury and activated CD8+ T cell infiltration in the ECM mouse brain. A) H&E staining of ECM brains showed multiple spots of intracerebral hemorrhage (dark blue arrow). B) IHC staining of synaptophysin (light blue arrow) in the cerebrum of control and ECM mice. C) Nissl staining of neurons (pink arrow) in the brainstem of control and ECM mice. Data are expressed as mean ± SD; n = 8 fields per group. D) IF staining of TUNEL+ cells in the olfactory bulb, cerebrum, cerebellum, and brainstem of control and ECM mice. E) IF staining of LC3 in neurons in the cerebrum of control and ECM mice. F) IF staining of Ki67+CD8+ T cells (yellow arrow) in the olfactory bulb of ECM mice. Fig. S2 The interaction of neurons and ECM CD8+ T cells in vitro. A) IF staining of naïve or ECM CD8+ T cells (yellow arrow) adhering to neurons (left image) and quantification of adhered CD8+ T cells (right image). Data are expressed as mean ± SD; unpaired t-test, n > 3 sections per group. B) CCK-8 detection in the supernatant of neurons treated with different proportions of CD8+ T cell culture supernatant. Data are expressed as mean ± SD; unpaired t-test, n = 4 per group. C) Flow cytometry of JC-1 (FL-1: monomer, FL-2: J-aggregates) in neurons co-cultured with ECM CD8+ T cell. D) IF staining of ECM CD8+ T cell (yellow arrow) adhering to axon. E) q-PCR detection of the H2-D1 expression in neurons co-cultured with ECM CD8+ T cell. Data are expressed as mean ± SD; unpaired t-test, n = 3 per group. F) Flow cytometry of the H2-D/K levels on neurons co-cultured with ECM CD8+ T cell. G) IF staining of H2-D/K and CD18 in CD8+ T cell (yellow arrow) and neuron (white arrow) co-culture system. Fig. S3 IFNβ or IFNγ induces neurons to upregulate PD-L1. A) IHC staining of PD-L1 in the olfactory bulb, cerebrum, and cerebellum of control and ECM mice (red arrow: PD-L1+ nerve cells). B) q-PCR analysis of Cd274 expression in neurons with IFNβ (100 U/mL, the same below) or [file 12974_2024_3114_MOESM1_ESM.zip › Supplementary figure1-6/Figure S3 ╨í╫╓║┼.png]

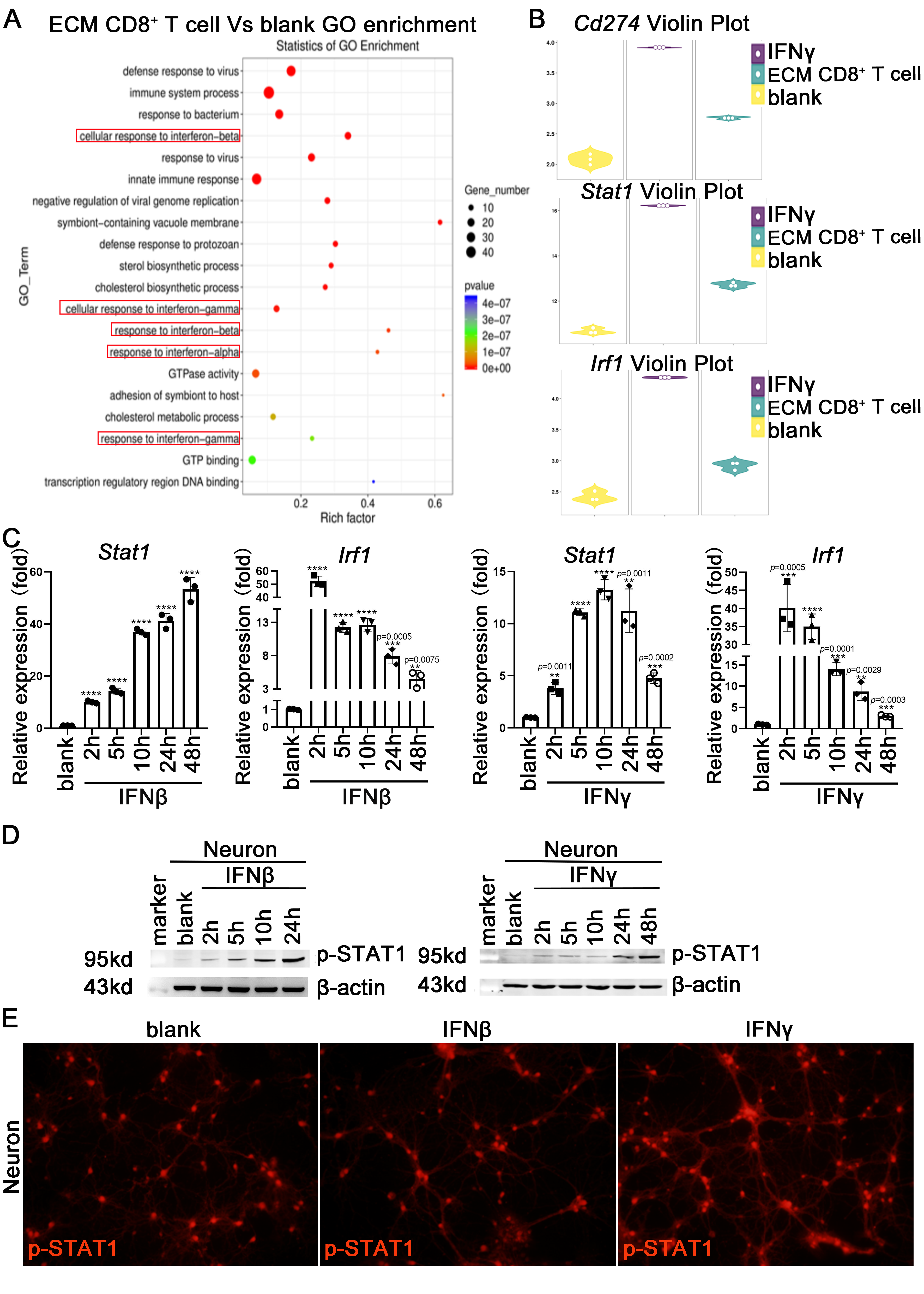

Supplement: Supplementary file 1 — Supplementary Material 1: Fig. S1. Nerve cell injury and activated CD8+ T cell infiltration in the ECM mouse brain. A) H&E staining of ECM brains showed multiple spots of intracerebral hemorrhage (dark blue arrow). B) IHC staining of synaptophysin (light blue arrow) in the cerebrum of control and ECM mice. C) Nissl staining of neurons (pink arrow) in the brainstem of control and ECM mice. Data are expressed as mean ± SD; n = 8 fields per group. D) IF staining of TUNEL+ cells in the olfactory bulb, cerebrum, cerebellum, and brainstem of control and ECM mice. E) IF staining of LC3 in neurons in the cerebrum of control and ECM mice. F) IF staining of Ki67+CD8+ T cells (yellow arrow) in the olfactory bulb of ECM mice. Fig. S2 The interaction of neurons and ECM CD8+ T cells in vitro. A) IF staining of naïve or ECM CD8+ T cells (yellow arrow) adhering to neurons (left image) and quantification of adhered CD8+ T cells (right image). Data are expressed as mean ± SD; unpaired t-test, n > 3 sections per group. B) CCK-8 detection in the supernatant of neurons treated with different proportions of CD8+ T cell culture supernatant. Data are expressed as mean ± SD; unpaired t-test, n = 4 per group. C) Flow cytometry of JC-1 (FL-1: monomer, FL-2: J-aggregates) in neurons co-cultured with ECM CD8+ T cell. D) IF staining of ECM CD8+ T cell (yellow arrow) adhering to axon. E) q-PCR detection of the H2-D1 expression in neurons co-cultured with ECM CD8+ T cell. Data are expressed as mean ± SD; unpaired t-test, n = 3 per group. F) Flow cytometry of the H2-D/K levels on neurons co-cultured with ECM CD8+ T cell. G) IF staining of H2-D/K and CD18 in CD8+ T cell (yellow arrow) and neuron (white arrow) co-culture system. Fig. S3 IFNβ or IFNγ induces neurons to upregulate PD-L1. A) IHC staining of PD-L1 in the olfactory bulb, cerebrum, and cerebellum of control and ECM mice (red arrow: PD-L1+ nerve cells). B) q-PCR analysis of Cd274 expression in neurons with IFNβ (100 U/mL, the same below) or [file 12974_2024_3114_MOESM1_ESM.zip › Supplementary figure1-6/Figure S4 A-E ╨í╫╓║┼.png]

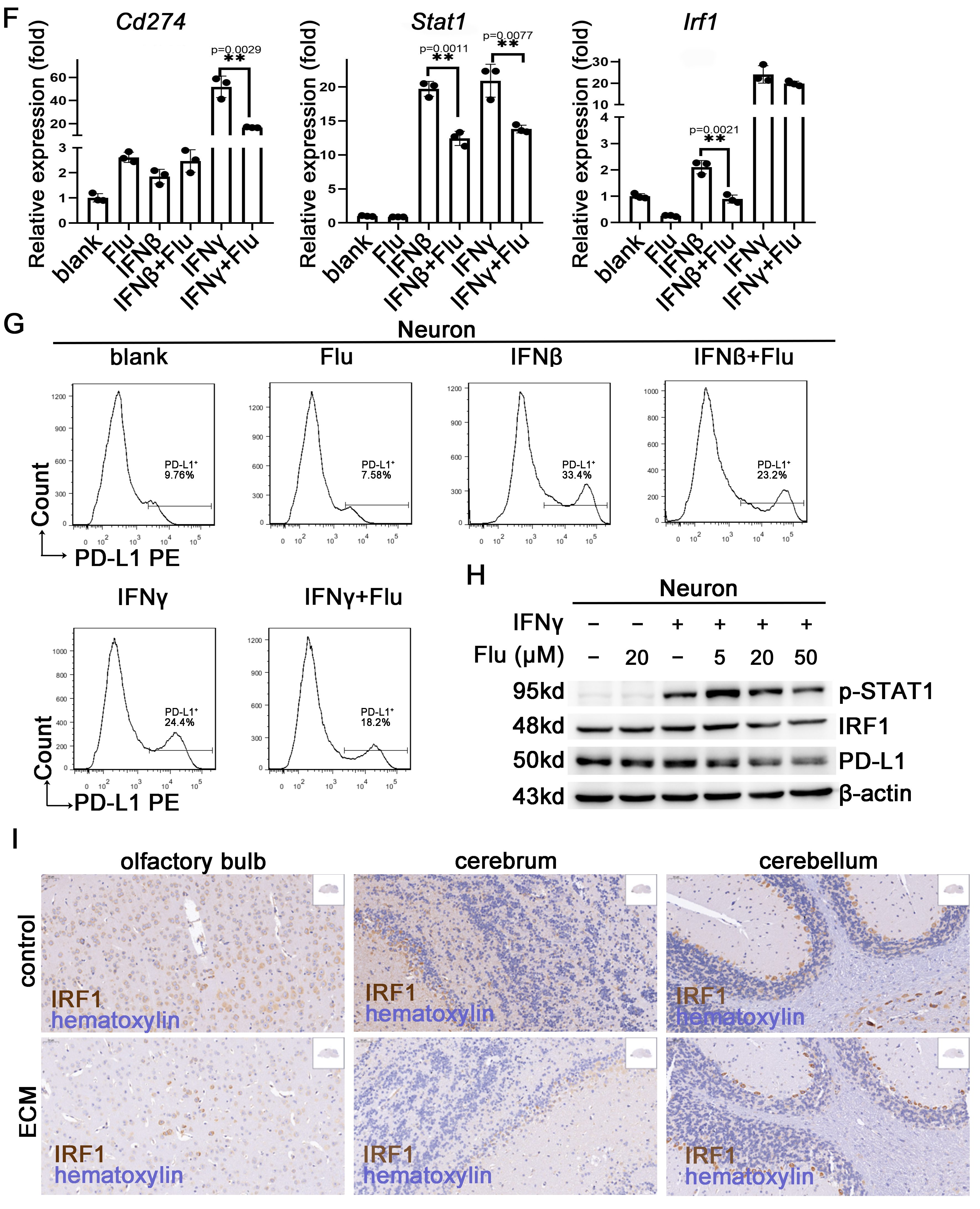

Supplement: Supplementary file 1 — Supplementary Material 1: Fig. S1. Nerve cell injury and activated CD8+ T cell infiltration in the ECM mouse brain. A) H&E staining of ECM brains showed multiple spots of intracerebral hemorrhage (dark blue arrow). B) IHC staining of synaptophysin (light blue arrow) in the cerebrum of control and ECM mice. C) Nissl staining of neurons (pink arrow) in the brainstem of control and ECM mice. Data are expressed as mean ± SD; n = 8 fields per group. D) IF staining of TUNEL+ cells in the olfactory bulb, cerebrum, cerebellum, and brainstem of control and ECM mice. E) IF staining of LC3 in neurons in the cerebrum of control and ECM mice. F) IF staining of Ki67+CD8+ T cells (yellow arrow) in the olfactory bulb of ECM mice. Fig. S2 The interaction of neurons and ECM CD8+ T cells in vitro. A) IF staining of naïve or ECM CD8+ T cells (yellow arrow) adhering to neurons (left image) and quantification of adhered CD8+ T cells (right image). Data are expressed as mean ± SD; unpaired t-test, n > 3 sections per group. B) CCK-8 detection in the supernatant of neurons treated with different proportions of CD8+ T cell culture supernatant. Data are expressed as mean ± SD; unpaired t-test, n = 4 per group. C) Flow cytometry of JC-1 (FL-1: monomer, FL-2: J-aggregates) in neurons co-cultured with ECM CD8+ T cell. D) IF staining of ECM CD8+ T cell (yellow arrow) adhering to axon. E) q-PCR detection of the H2-D1 expression in neurons co-cultured with ECM CD8+ T cell. Data are expressed as mean ± SD; unpaired t-test, n = 3 per group. F) Flow cytometry of the H2-D/K levels on neurons co-cultured with ECM CD8+ T cell. G) IF staining of H2-D/K and CD18 in CD8+ T cell (yellow arrow) and neuron (white arrow) co-culture system. Fig. S3 IFNβ or IFNγ induces neurons to upregulate PD-L1. A) IHC staining of PD-L1 in the olfactory bulb, cerebrum, and cerebellum of control and ECM mice (red arrow: PD-L1+ nerve cells). B) q-PCR analysis of Cd274 expression in neurons with IFNβ (100 U/mL, the same below) or [file 12974_2024_3114_MOESM1_ESM.zip › Supplementary figure1-6/Figure S4 F-I ╨í╫╓║┼.png]

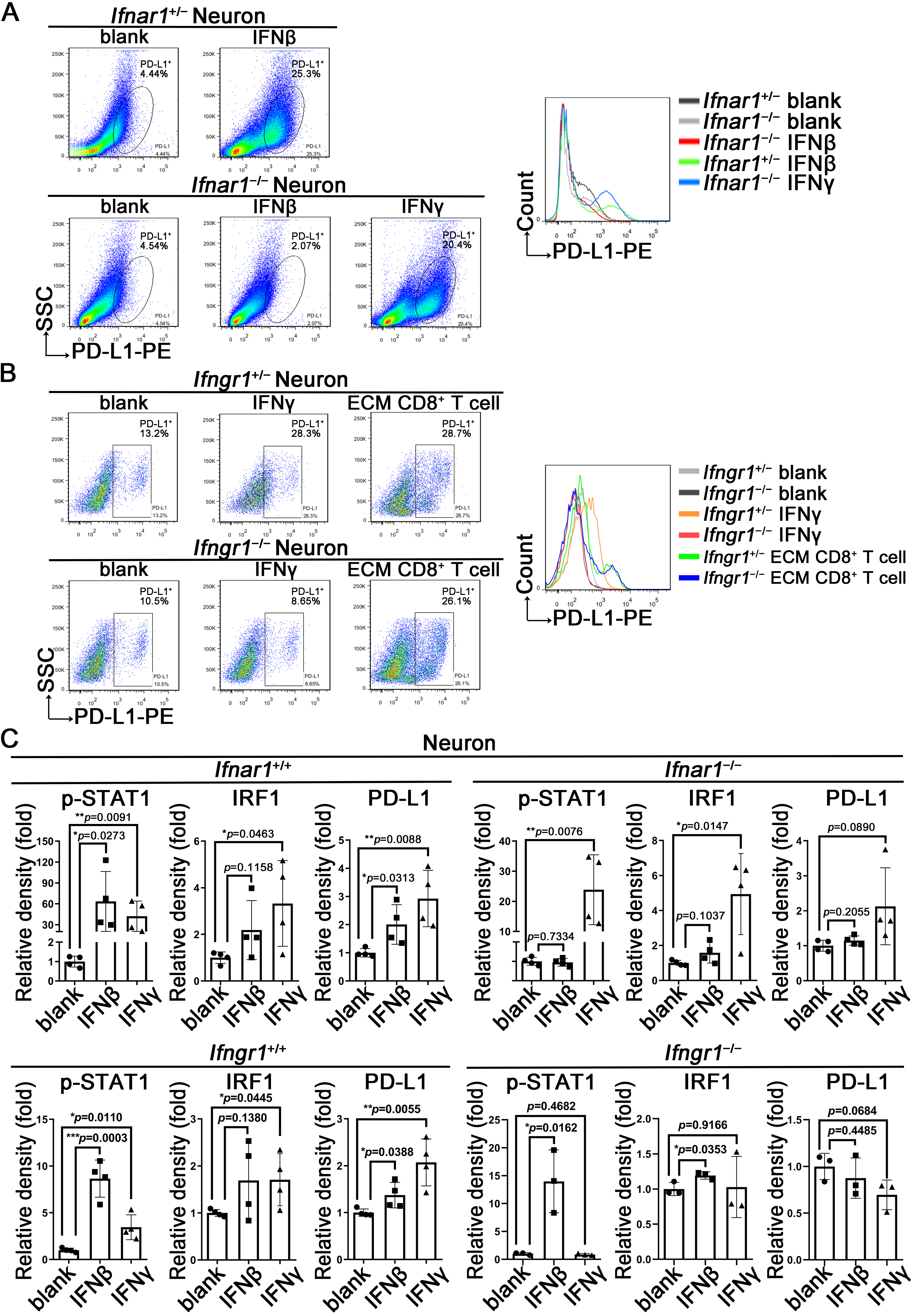

Supplement: Supplementary file 1 — Supplementary Material 1: Fig. S1. Nerve cell injury and activated CD8+ T cell infiltration in the ECM mouse brain. A) H&E staining of ECM brains showed multiple spots of intracerebral hemorrhage (dark blue arrow). B) IHC staining of synaptophysin (light blue arrow) in the cerebrum of control and ECM mice. C) Nissl staining of neurons (pink arrow) in the brainstem of control and ECM mice. Data are expressed as mean ± SD; n = 8 fields per group. D) IF staining of TUNEL+ cells in the olfactory bulb, cerebrum, cerebellum, and brainstem of control and ECM mice. E) IF staining of LC3 in neurons in the cerebrum of control and ECM mice. F) IF staining of Ki67+CD8+ T cells (yellow arrow) in the olfactory bulb of ECM mice. Fig. S2 The interaction of neurons and ECM CD8+ T cells in vitro. A) IF staining of naïve or ECM CD8+ T cells (yellow arrow) adhering to neurons (left image) and quantification of adhered CD8+ T cells (right image). Data are expressed as mean ± SD; unpaired t-test, n > 3 sections per group. B) CCK-8 detection in the supernatant of neurons treated with different proportions of CD8+ T cell culture supernatant. Data are expressed as mean ± SD; unpaired t-test, n = 4 per group. C) Flow cytometry of JC-1 (FL-1: monomer, FL-2: J-aggregates) in neurons co-cultured with ECM CD8+ T cell. D) IF staining of ECM CD8+ T cell (yellow arrow) adhering to axon. E) q-PCR detection of the H2-D1 expression in neurons co-cultured with ECM CD8+ T cell. Data are expressed as mean ± SD; unpaired t-test, n = 3 per group. F) Flow cytometry of the H2-D/K levels on neurons co-cultured with ECM CD8+ T cell. G) IF staining of H2-D/K and CD18 in CD8+ T cell (yellow arrow) and neuron (white arrow) co-culture system. Fig. S3 IFNβ or IFNγ induces neurons to upregulate PD-L1. A) IHC staining of PD-L1 in the olfactory bulb, cerebrum, and cerebellum of control and ECM mice (red arrow: PD-L1+ nerve cells). B) q-PCR analysis of Cd274 expression in neurons with IFNβ (100 U/mL, the same below) or [file 12974_2024_3114_MOESM1_ESM.zip › Supplementary figure1-6/Figure S5 A-C ╨í╫╓║┼╕─SD sy╕─3-17.png]

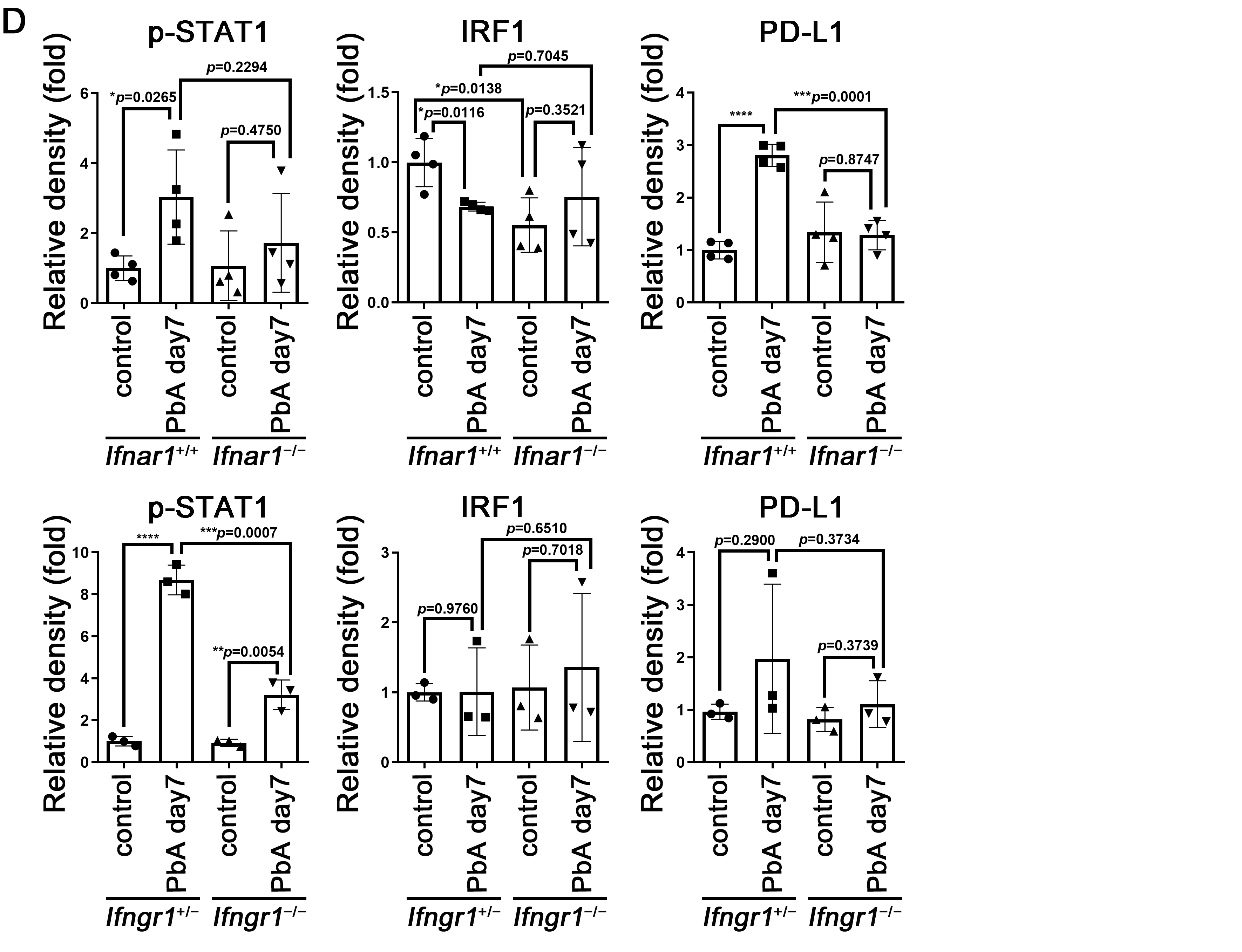

Supplement: Supplementary file 1 — Supplementary Material 1: Fig. S1. Nerve cell injury and activated CD8+ T cell infiltration in the ECM mouse brain. A) H&E staining of ECM brains showed multiple spots of intracerebral hemorrhage (dark blue arrow). B) IHC staining of synaptophysin (light blue arrow) in the cerebrum of control and ECM mice. C) Nissl staining of neurons (pink arrow) in the brainstem of control and ECM mice. Data are expressed as mean ± SD; n = 8 fields per group. D) IF staining of TUNEL+ cells in the olfactory bulb, cerebrum, cerebellum, and brainstem of control and ECM mice. E) IF staining of LC3 in neurons in the cerebrum of control and ECM mice. F) IF staining of Ki67+CD8+ T cells (yellow arrow) in the olfactory bulb of ECM mice. Fig. S2 The interaction of neurons and ECM CD8+ T cells in vitro. A) IF staining of naïve or ECM CD8+ T cells (yellow arrow) adhering to neurons (left image) and quantification of adhered CD8+ T cells (right image). Data are expressed as mean ± SD; unpaired t-test, n > 3 sections per group. B) CCK-8 detection in the supernatant of neurons treated with different proportions of CD8+ T cell culture supernatant. Data are expressed as mean ± SD; unpaired t-test, n = 4 per group. C) Flow cytometry of JC-1 (FL-1: monomer, FL-2: J-aggregates) in neurons co-cultured with ECM CD8+ T cell. D) IF staining of ECM CD8+ T cell (yellow arrow) adhering to axon. E) q-PCR detection of the H2-D1 expression in neurons co-cultured with ECM CD8+ T cell. Data are expressed as mean ± SD; unpaired t-test, n = 3 per group. F) Flow cytometry of the H2-D/K levels on neurons co-cultured with ECM CD8+ T cell. G) IF staining of H2-D/K and CD18 in CD8+ T cell (yellow arrow) and neuron (white arrow) co-culture system. Fig. S3 IFNβ or IFNγ induces neurons to upregulate PD-L1. A) IHC staining of PD-L1 in the olfactory bulb, cerebrum, and cerebellum of control and ECM mice (red arrow: PD-L1+ nerve cells). B) q-PCR analysis of Cd274 expression in neurons with IFNβ (100 U/mL, the same below) or [file 12974_2024_3114_MOESM1_ESM.zip › Supplementary figure1-6/Figure S5 D ╨í╫╓║┼╕─SD.png]

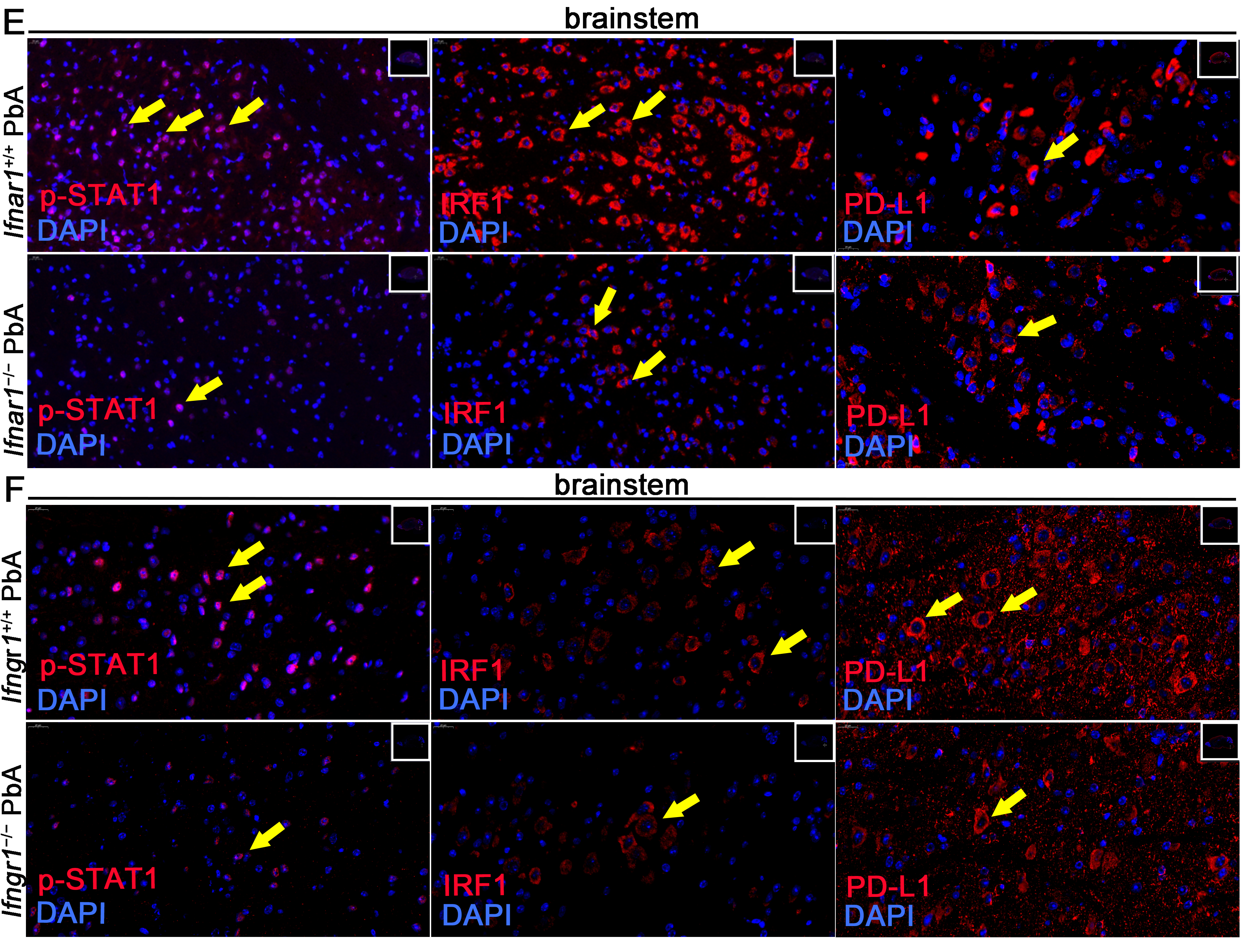

Supplement: Supplementary file 1 — Supplementary Material 1: Fig. S1. Nerve cell injury and activated CD8+ T cell infiltration in the ECM mouse brain. A) H&E staining of ECM brains showed multiple spots of intracerebral hemorrhage (dark blue arrow). B) IHC staining of synaptophysin (light blue arrow) in the cerebrum of control and ECM mice. C) Nissl staining of neurons (pink arrow) in the brainstem of control and ECM mice. Data are expressed as mean ± SD; n = 8 fields per group. D) IF staining of TUNEL+ cells in the olfactory bulb, cerebrum, cerebellum, and brainstem of control and ECM mice. E) IF staining of LC3 in neurons in the cerebrum of control and ECM mice. F) IF staining of Ki67+CD8+ T cells (yellow arrow) in the olfactory bulb of ECM mice. Fig. S2 The interaction of neurons and ECM CD8+ T cells in vitro. A) IF staining of naïve or ECM CD8+ T cells (yellow arrow) adhering to neurons (left image) and quantification of adhered CD8+ T cells (right image). Data are expressed as mean ± SD; unpaired t-test, n > 3 sections per group. B) CCK-8 detection in the supernatant of neurons treated with different proportions of CD8+ T cell culture supernatant. Data are expressed as mean ± SD; unpaired t-test, n = 4 per group. C) Flow cytometry of JC-1 (FL-1: monomer, FL-2: J-aggregates) in neurons co-cultured with ECM CD8+ T cell. D) IF staining of ECM CD8+ T cell (yellow arrow) adhering to axon. E) q-PCR detection of the H2-D1 expression in neurons co-cultured with ECM CD8+ T cell. Data are expressed as mean ± SD; unpaired t-test, n = 3 per group. F) Flow cytometry of the H2-D/K levels on neurons co-cultured with ECM CD8+ T cell. G) IF staining of H2-D/K and CD18 in CD8+ T cell (yellow arrow) and neuron (white arrow) co-culture system. Fig. S3 IFNβ or IFNγ induces neurons to upregulate PD-L1. A) IHC staining of PD-L1 in the olfactory bulb, cerebrum, and cerebellum of control and ECM mice (red arrow: PD-L1+ nerve cells). B) q-PCR analysis of Cd274 expression in neurons with IFNβ (100 U/mL, the same below) or [file 12974_2024_3114_MOESM1_ESM.zip › Supplementary figure1-6/Figure S5 E-F ╨í╫╓║┼.png]

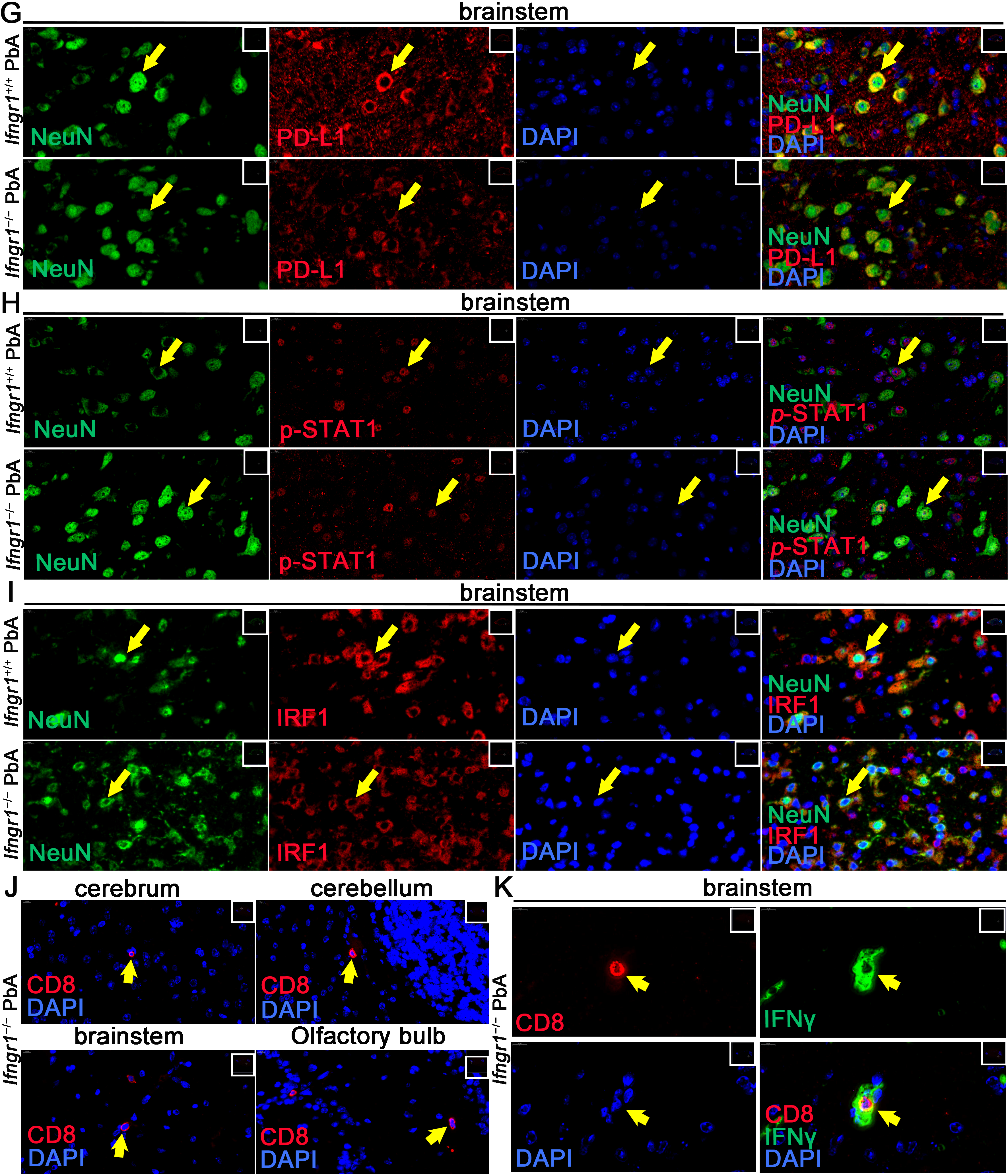

Supplement: Supplementary file 1 — Supplementary Material 1: Fig. S1. Nerve cell injury and activated CD8+ T cell infiltration in the ECM mouse brain. A) H&E staining of ECM brains showed multiple spots of intracerebral hemorrhage (dark blue arrow). B) IHC staining of synaptophysin (light blue arrow) in the cerebrum of control and ECM mice. C) Nissl staining of neurons (pink arrow) in the brainstem of control and ECM mice. Data are expressed as mean ± SD; n = 8 fields per group. D) IF staining of TUNEL+ cells in the olfactory bulb, cerebrum, cerebellum, and brainstem of control and ECM mice. E) IF staining of LC3 in neurons in the cerebrum of control and ECM mice. F) IF staining of Ki67+CD8+ T cells (yellow arrow) in the olfactory bulb of ECM mice. Fig. S2 The interaction of neurons and ECM CD8+ T cells in vitro. A) IF staining of naïve or ECM CD8+ T cells (yellow arrow) adhering to neurons (left image) and quantification of adhered CD8+ T cells (right image). Data are expressed as mean ± SD; unpaired t-test, n > 3 sections per group. B) CCK-8 detection in the supernatant of neurons treated with different proportions of CD8+ T cell culture supernatant. Data are expressed as mean ± SD; unpaired t-test, n = 4 per group. C) Flow cytometry of JC-1 (FL-1: monomer, FL-2: J-aggregates) in neurons co-cultured with ECM CD8+ T cell. D) IF staining of ECM CD8+ T cell (yellow arrow) adhering to axon. E) q-PCR detection of the H2-D1 expression in neurons co-cultured with ECM CD8+ T cell. Data are expressed as mean ± SD; unpaired t-test, n = 3 per group. F) Flow cytometry of the H2-D/K levels on neurons co-cultured with ECM CD8+ T cell. G) IF staining of H2-D/K and CD18 in CD8+ T cell (yellow arrow) and neuron (white arrow) co-culture system. Fig. S3 IFNβ or IFNγ induces neurons to upregulate PD-L1. A) IHC staining of PD-L1 in the olfactory bulb, cerebrum, and cerebellum of control and ECM mice (red arrow: PD-L1+ nerve cells). B) q-PCR analysis of Cd274 expression in neurons with IFNβ (100 U/mL, the same below) or [file 12974_2024_3114_MOESM1_ESM.zip › Supplementary figure1-6/Figure S5 G-K ╨í╫╓║┼.png]

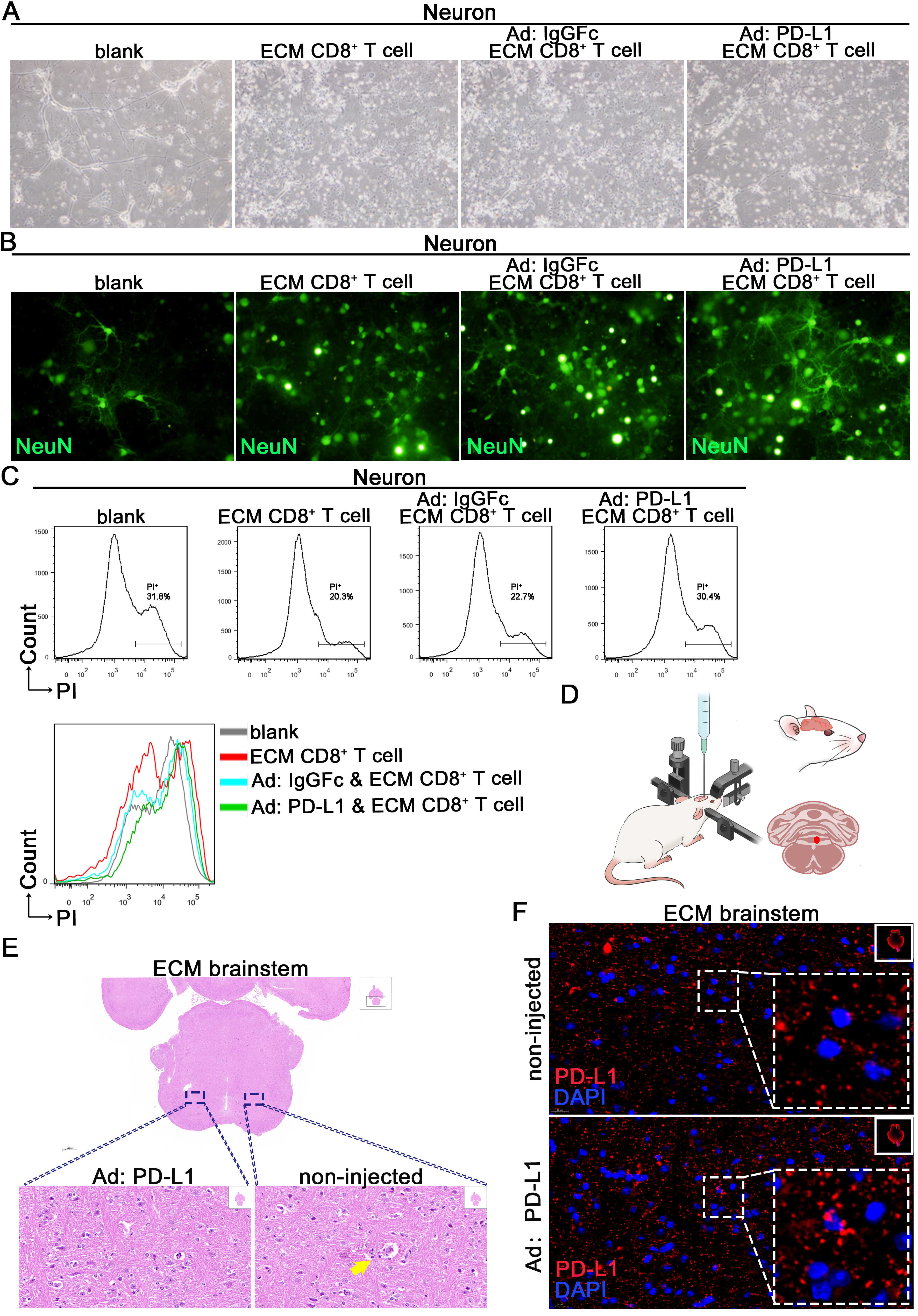

Supplement: Supplementary file 1 — Supplementary Material 1: Fig. S1. Nerve cell injury and activated CD8+ T cell infiltration in the ECM mouse brain. A) H&E staining of ECM brains showed multiple spots of intracerebral hemorrhage (dark blue arrow). B) IHC staining of synaptophysin (light blue arrow) in the cerebrum of control and ECM mice. C) Nissl staining of neurons (pink arrow) in the brainstem of control and ECM mice. Data are expressed as mean ± SD; n = 8 fields per group. D) IF staining of TUNEL+ cells in the olfactory bulb, cerebrum, cerebellum, and brainstem of control and ECM mice. E) IF staining of LC3 in neurons in the cerebrum of control and ECM mice. F) IF staining of Ki67+CD8+ T cells (yellow arrow) in the olfactory bulb of ECM mice. Fig. S2 The interaction of neurons and ECM CD8+ T cells in vitro. A) IF staining of naïve or ECM CD8+ T cells (yellow arrow) adhering to neurons (left image) and quantification of adhered CD8+ T cells (right image). Data are expressed as mean ± SD; unpaired t-test, n > 3 sections per group. B) CCK-8 detection in the supernatant of neurons treated with different proportions of CD8+ T cell culture supernatant. Data are expressed as mean ± SD; unpaired t-test, n = 4 per group. C) Flow cytometry of JC-1 (FL-1: monomer, FL-2: J-aggregates) in neurons co-cultured with ECM CD8+ T cell. D) IF staining of ECM CD8+ T cell (yellow arrow) adhering to axon. E) q-PCR detection of the H2-D1 expression in neurons co-cultured with ECM CD8+ T cell. Data are expressed as mean ± SD; unpaired t-test, n = 3 per group. F) Flow cytometry of the H2-D/K levels on neurons co-cultured with ECM CD8+ T cell. G) IF staining of H2-D/K and CD18 in CD8+ T cell (yellow arrow) and neuron (white arrow) co-culture system. Fig. S3 IFNβ or IFNγ induces neurons to upregulate PD-L1. A) IHC staining of PD-L1 in the olfactory bulb, cerebrum, and cerebellum of control and ECM mice (red arrow: PD-L1+ nerve cells). B) q-PCR analysis of Cd274 expression in neurons with IFNβ (100 U/mL, the same below) or [file 12974_2024_3114_MOESM1_ESM.zip › Supplementary figure1-6/Figure S6 ╨í╫╓║┼3-15╬─╒┬╙├.png]
