# Supplementary material for: Neurons upregulate PD-L1 via IFN/STAT1/IRF1 to alleviate damage by CD8+ T cells in cerebral malaria
Source: J Neuroinflammation. 2024 May 7;21:119. doi: 10.1186/s12974-024-03114-7 (PMC11077882; doi:10.1186/s12974-024-03114-7)
Supplement: Supplementary file 2 — Supplementary Material 2: Table S1. List of primer sequences used for q-PCR. [file 12974_2024_3114_MOESM2_ESM.docx]

Table S1 Primer sequences used in q-PCR

| *Cd274* |  |
| --- | --- |
| mPD-L1 F  mPD-L1 R | AGTATGGCAGCAACGTCACG  TCCTTTTCCCAGTACACCACTA |
| *H2-D1* |  |
| mMHCI H-2D F  mMHCI H-2D R | GATGCAGAGCATTACAGGGC  GCCAGGTCAGGGCAATGTC |
| *Stat1* |  |
| mStat1 F  mStat1 R | GAGAACACCCCTTCTACCACT  GCCTCTGTAATCACTCATCACGA |
| *Irf1* |  |
| mIRF-1 F  mIRF-1 R | ATGCCAATCACTCGAATGCG  CCTGCTTTGTATCGGCCTGT |
| *β-actin* |  |
| β-actin F  β-actin R | CATCCGTAAAGACCTCTATGCCAA C  ATGGAGCCACCGATCCACA |
